# SHQ1 regulation of RNA splicing is required for T-lymphoblastic leukemia cell survival

**DOI:** 10.1038/s41467-018-06523-4

**Published:** 2018-10-15

**Authors:** Hexiu Su, Juncheng Hu, Liang Huang, Yang Yang, Morgan Thenoz, Anna Kuchmiy, Yufeng Hu, Peng Li, Hui Feng, Yu Zhou, Tom Taghon, Pieter Van Vlierberghe, Guoliang Qing, Zhichao Chen, Hudan Liu

**Affiliations:** 1 Institute of Hematology, Union Hospital, Tongji Medical College, Huazhong University of Science and Technology, Wuhan, 430022 China; 2 Medical Research Institute, Wuhan University, Wuhan, 430071 China; 3 Department of Hematology, Tongji Hospital, Tongji Medical College, Huazhong University of Science and Technology, Wuhan, 430030 China; 4 College of Life Sciences, Wuhan University, Wuhan, 430072 China; 5 Department of Biomolecular Medicine, Ghent University, Ghent, 9000 Belgium; 6 Department of Clinical Chemistry, Microbiology and Immunology, Ghent University, Ghent, 9000 Belgium; 7 Zhongnan Hospital of Wuhan University, Wuhan, 430071 China; 8 South China Institute for Stem Cell Biology and Regenerative Medicine, Guangzhou Institutes of Biomedicine and Health, Chinese Academy of Sciences, Guangzhou, 510530 China; 9 Department of Pharmacology and Department of Medicine, Cancer Research Center, Section of Hematology and Medical Oncology, Boston University School of Medicine, Boston, MA 02118 USA

## Abstract

T-acute lymphoblastic leukemia (T-ALL) is an aggressive hematologic malignancy with complicated heterogeneity. Although expression profiling reveals common elevated genes in distinct T-ALL subtypes, little is known about their functional role(s) and regulatory mechanism(s). We here show that SHQ1, an H/ACA snoRNP assembly factor involved in snRNA pseudouridylation, is highly expressed in T-ALL. Mechanistically, oncogenic NOTCH1 directly binds to the *SHQ1* promoter and activates its transcription. *SHQ1* depletion induces T-ALL cell death in vitro and prolongs animal survival in murine T-ALL models. RNA-Seq reveals that *SHQ1* depletion impairs widespread RNA splicing, and *MYC* is one of the most prominently downregulated genes due to inefficient splicing. *MYC* overexpression significantly rescues T-ALL cell death resulted from *SHQ1* inactivation. We herein report a mechanism of NOTCH1–SHQ1–MYC axis in T-cell leukemogenesis. These findings not only shed light on the role of SHQ1 in RNA splicing and tumorigenesis, but also provide additional insight into *MYC* regulation.

## Abstract (web summary)

T-acute lymphoblastic leukemia is an aggressive cancer. Here the authors provide insights into the functional role of SHQ1, an H/ACA snoRNP assembly factor involved in snRNA pseudouridylation, in T-lymphoblastic leukemia cell survival through regulating the maturation of MYC mRNA.

## Introduction

T-cell acute lymphoblastic leukemia (T-ALL) is a lethal and aggressive hematological malignancy that frequently affects children and adolescents, and accounts for approximately 10–15% of newly diagnosed pediatric ALL. Although clinical complete remission is approaching 90% due to the implementation of intensive chemotherapy protocols, the outcomes of patients with relapsed or refractory T-ALL remain poor, with cure rates of less than 40%^1^. This clinical challenge has fueled considerable research into the molecular understanding of T-ALL pathogenesis which has yielded immense progress in the past decade^2^. Gene expression profiling of T-ALL cases has led to the identification of subgroups of T-ALL, each characterized by aberrant expression of one particular transcription factor such as TAL1, TLX1, and LMO1/2^3,4^. Genome-wide sequencing has identified numerous somatic gene mutations in T-ALL, in which *NOTCH1* gain-of-function mutations are found in >50% of T-ALL cases^5^ and *FBW7*, the gene encoding the NOTCH1 E3 ligase, is mutated with impaired activity at the rate of 12%^6,7^. These findings have vaulted the dysregulated NOTCH1 signaling to the center of T-ALL pathogenesis^8^.

Activation of NOTCH1 signaling initiates with association to ligands of the Delta/Serrate/Lag-2 (DSL) family on neighboring cells. This interaction elicits a series of proteolytic cleavage terminated by γ-secretase. As a result, intracellular NOTCH1 (ICN1) is released from the cell membrane and translocates into the nucleus, activating downstream responder genes by forming a transcriptional activation complex^9^. Persistent activation of NOTCH1 signaling, due to *NOTCH1* gain-of-function and/or *FBW7* loss-of-function mutations, triggers overexpression of multiple oncogenes in T-ALL. *MYC* has been demonstrated as a major downstream target of NOTCH1 which plays an essential role in T-cell leukemogenesis^10–12^.

Pseudouridine (Ψ), a C5-glycoside isomer of uridine, is the most abundant posttranscriptional modification in cellular RNAs^13^. Pseudouridines in ribosomal RNA (rRNA) and small nuclear (snRNA) are essential for the correct function of the ribosome and spliceosome^14,15^. In higher eukaryotes, pseudouridylation is mainly governed by a family of box H/ACA snoRNPs (small nucleolar ribonucleoproteins), consisting of a unique box H/ACA snoRNA and four common core proteins (Cbf5/NAP57/Dyskerin, Nhp2/L7Ae, Nop10, and Gar1). The RNA component serves as a guide that base pairs with the substrate RNA and directs the enzyme Cbf5 to carry out the pseudouridylation reaction at a specific site^16^. During the assembly process of H/ACA snoRNPs, SHQ1 functions as an assembly chaperone that protects the Cbf5 protein complexes from non-specific RNA binding and aggregation before its assembly with H/ACA snoRNA^17^. As such, abrogation of SHQ1 activity results in assembly failure and loss of H/ACA snoRNP function^18,19^. Despite well-documented mechanism of SHQ1 in H/ACA snoRNP biogenesis, little is known about its precise functional role, especially in human diseases such as cancer.

We herein define a vital role of SHQ1 in supporting T-cell leukemogenesis. Sustained *SHQ1* expression, induced by oncogenic NOTCH1, is essential for T-ALL cell growth in vitro and leukemogenesis in vivo. The profound role of SHQ1 in leukemogenesis relies on successful H/ACA snoRNP assembly, enabling efficient global pre-mRNA splicing. We also identify *MYC*, whose splicing and expression are highly dependent on SHQ1, as an important downstream effector mediating the tumor-supporting role of SHQ1. These findings provide important insights into how SHQ1-mediated RNA modification and pre-mRNA splicing affect tumorigenesis, and also deepen our understanding of posttranscriptional regulation of oncogene *MYC*.

## Results

### SHQ1 is highly expressed in T-ALL

**Fig. 1:**
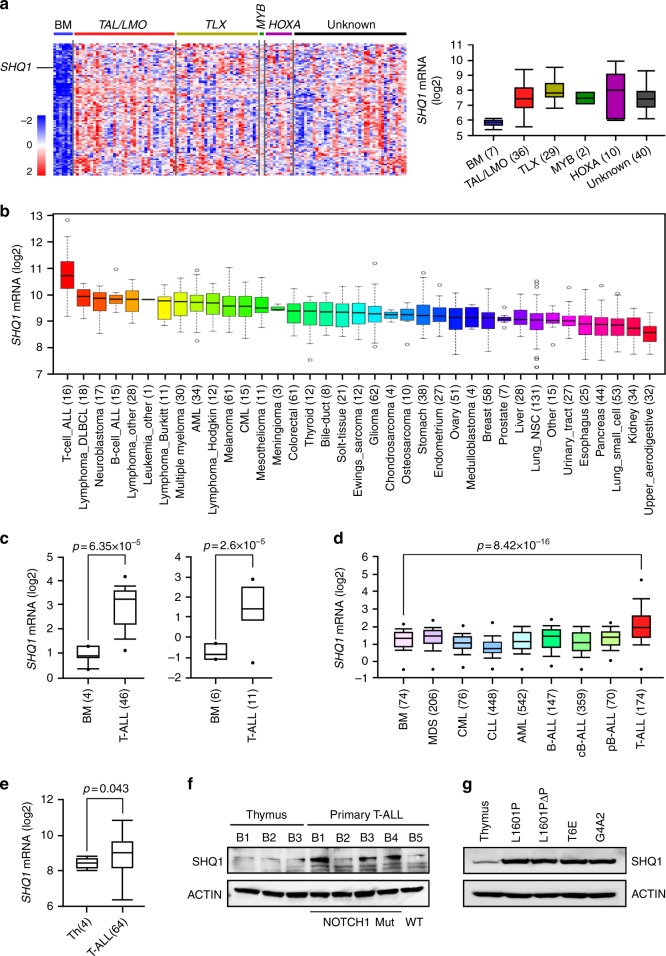
Specific high SHQ1 expression in T-ALL. **a** Heatmap of top 97 highly expressed genes in 117 pediatric T-ALL samples in comparison to 7 normal bone marrow cells (BM). Unsupervised hierarchical cluster was analyzed from GSE26713. A total of 77 T-ALL samples are characterized by oncogenic rearrangements, including *TAL/LMO* (*n* = 36), *TLX* (*n* = 29), *MYB* translocations (*n* = 2), and *HOXA* activating rearrangements (*n* = 10). Samples without such abnormalities are identified as Unknown (*n* = 40). Average gene expression from each category of T-ALL was compared to that in normal BM. Common genes highly expressed in T-ALL are clustered and shown in a descending order as to gene expression levels in T-ALL (left; red indicates increased and blue decreased) (see Supplementary Data 1). Distributions of *SHQ1* mRNA expression derived from the heatmap are presented on the right. **b**, **c** *SHQ1* expression was analyzed among 1036 human cancer cell lines in CCLE database (https://portals.broadinstitute.org/ccle) (**b**), as well as other primary T-ALLs and normal BM (left, 46 T-ALL samples in GSE28497; right, 11 T-ALL samples in GSE7186) in (**c**). **d** Expression analysis of *SHQ1* among 2096 primary samples of hematological diseases and BM (GSE13159). **e** *SHQ1* expression was analyzed from gene expression profiling of 64 human T-ALL samples and 4 normal CD4^+^CD8^+^ thymocyte samples. Above all, sample numbers are shown in parentheses. The distributions of *SHQ1* mRNA expression are presented as log2 median-entered intensity and shown in box-and-whisker plots with the median value (line), the interquartile range (box), and up to 1.5× the interquartile range (bars). Unpaired *t-*test was used for statistic analysis in (**c**–**e**). **f** Immunoblots of SHQ1 in 3 normal thymus and 5 primary T-ALL patient samples. *NOTCH1* mutation status in primary T-ALL is also provided. These samples were obtained from Belgium and labeled as B1–B5. **g** Immunoblots of murine SHQ1 in normal C57BL/6 thymus cells as well as 2 primary murine T-ALL samples Kras^G12D^/NOTCH1^L1601P^ (L1601P), Kras^G12D^/NOTCH1^L1601PΔP^ (L1601PΔP), and 2 murine T-ALL cell lines T6E and G4A2. ACTIN serves as a loading control

To screen common elevated genes with tumorigenic potential, we compared gene expression profiles of 117 diagnostic pediatric T-ALLs with 7 normal bone marrow (BM) controls^20^. A total of 97 genes were enriched with more than 1.5-fold upregulation in T-ALL (*p* < 0.01, unpaired *t*-test). One notable finding in this gene cluster was *SHQ1*, encoding an essential factor involved in H/ACA snoRNP biogenesis and RNA splicing (Fig. 1a and Supplementary Data 1). Analysis of the Cancer Cell Line Encyclopedia (CCLE) demonstrated *SHQ1* is most highly expressed in T-ALL among 1036 human cancer cell lines^21^ (Fig. 1b). Assessment of multiple human leukemia databases confirmed significant increase in *SHQ1* expression in T-ALL as compared to normal BM^22,23^ (Fig. 1c) or other hematological malignancies^24^ (Fig. 1d). In addition, previously published genome-wide expression profiling data from normal and malignant T cells^25^ confirmed significant higher *SHQ1* expression in primary human T-ALL (*n* = 64) as compared to CD4^+^CD8^+^ normal human thymocytes (Fig. 1e). Furthermore, primary T-ALL cells harboring *NOTCH1* activating mutations showed higher SHQ1 protein expression than a T-ALL case with wild-type *NOTCH1* or normal thymocytes (Fig. 1f). In line with the observations in human T-ALL, murine T-ALL cells with *NOTCH1* activating mutations/truncations had greater SHQ1 expression than normal thymocytes (Fig. 1g). Taken together, we identify a global upregulation of SHQ1 in T-ALL.

### NOTCH1 directly activates *SHQ1* expression in T-ALL

**Fig. 2:**
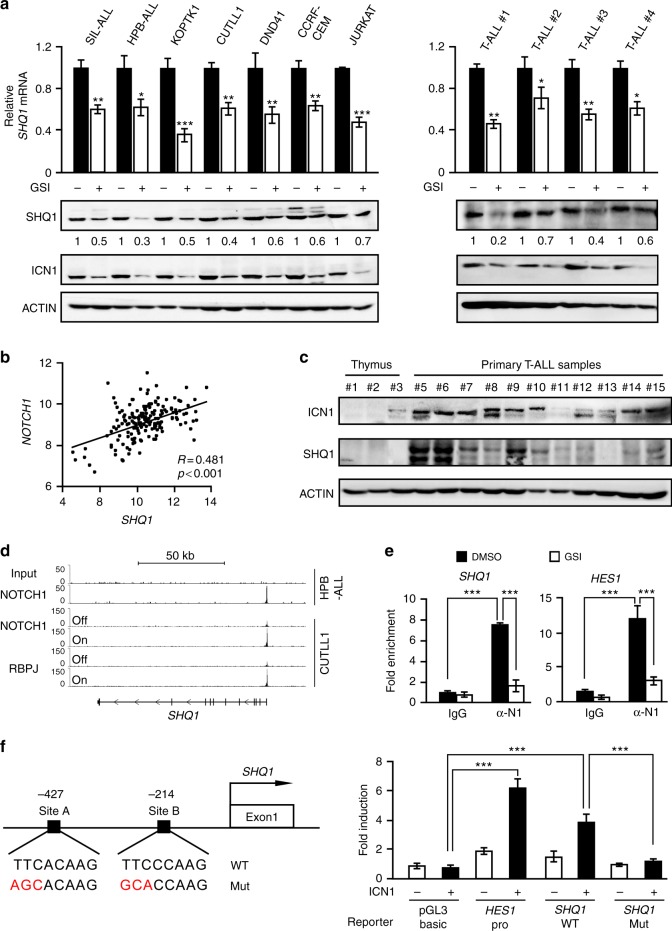
Intracellular NOTCH1 directly binds to and activates *SHQ1*. **a** Seven T-ALL cell lines and four primary T-ALL cells were subjected to GSI (Compound E, 1 μM) or DMSO treatment for 24 h. SHQ1 mRNA and protein levels were subsequently determined by quantitative polymerase chain reaction (qPCR) and immunoblots. **b** Correlation of *SHQ1* expression with *NOTCH1* in 174 primary T-ALL samples (GSE13159) with mRNA levels presented as log2 median-entered intensity. Pearson’s correlation coefficient (*R*) = 0.481, *p* < 0.001, paired *t*-test. **c** Immunoblots of SHQ1 and ICN1 in additional 11 primary T-ALL and 3 normal thymus samples. **d** Chromatin landscapes around the *SHQ1* locus in HPB-ALL (GSE58406) and CUTLL1 (GSE51800) cells. The associations of nuclear NOTCH1 and RBPJ with the *SHQ1* transcriptional start region are shown with respect to NOTCH1 active or inactive state. **e** Binding of ICN1 to the *SHQ1* or *HES1* promoter was analyzed by ChIP in SIL-ALL cells with or without GSI treatment (Compound E, 1 μM) for 24 h. Averages of fold enrichment between ICN1 and isotype IgG are shown. α-N1 denotes antibodies against ICN1. **f** Schematic presentation of two potential RBPJ-binding sites proximal to the *SHQ1* transcription initiation site. Luciferase assays were carried out with *SHQ1* DNA sequence (−441 to −194) or *HES1* promoter cloned into pGL3-basic vector; reporter activities relative to empty pGL3 vector without ICN1 are presented as average fold induction. Data shown represent the means ( ± SEM) of three biological replicates; *p < 0.05, **p < 0.01, ***p < 0.001, unpaired *t*-test (**a**, **e**, **f**)

To understand the molecular mechanism underlying SHQ1 upregulation in T-ALL, we performed in silico analysis to identify potential transcription factor binding *cis*-elements in the *SHQ1* locus. NOTCH1, GATA3, TAL1 and MYC were predicted to activate the *SHQ1* promoter (Supplementary Fig. 1a). We individually knocked down each of these transcription factors in human T-ALL JURKAT cells and found that only depletion of NOTCH1 induced SHQ1 downregulation (Supplementary Fig. 1b-1f). We then blocked NOTCH signaling using γ-secretase inhibitor (GSI) Compound E in T-ALL cells. As shown in Fig. 2a, NOTCH inactivation decreased *SHQ1* mRNA and protein levels in seven T-ALL cell lines and four primary patient-derived T-ALL cells. Again, genetic inactivation of NOTCH by expression of dominant negative MAML (DNMAML) in HPB-ALL cells diminished *SHQ1* mRNA (Supplementary Fig. 2a). Conversely, overexpression of intracellular NOTCH1 in either human HPB-ALL or murine T6E cells remarkably bolstered *SHQ1* expression in the presence of NOTCH inhibitor (Supplementary Fig. 2b). Moreover, gene expression profiling in 174 primary T-ALLs revealed a strong and significant correlation between *SHQ1* and NOTCH1-regulated signature genes including *NOTCH1* itself (Fig. 2b) as well as *NOTCH3* or *LZTFL1*^26^ (Supplementary Fig. 2c). Using three individual normal thymus specimens as control, we further verified these findings in 11 Chinese T-ALL patient samples, and found that SHQ1 was generally more abundant in leukemia cells with enhanced NOTCH1 activation, as judged by ICN1 production (Fig. 2c). These data support the presence of NOTCH1–SHQ1 axis in human T-ALL.

Importantly, removal of Compound E immediately reversed GSI-mediated inhibition of *SHQ1* expression, even in the presence of protein synthesis inhibitor cycloheximide (Supplementary Fig. 2d), suggesting a direct NOTCH1 transcriptional activation of *SHQ1*. In support of this prediction, chromatin immunoprecipitation-sequencing (ChIP-Seq) analysis revealed a NOTCH1 binding signal proximal to the *SHQ1* transcription start site^27,28^. The association of NOTCH1 was completely abolished in the NOTCH off state. Similarly, NOTCH1 inhibition compromised the binding of RBPJ, an essential component of the NOTCH1 transcriptional complex, to the *SHQ1* promoter (Fig. 2d). Conventional ChIP validated NOTCH1 association with the *SHQ1* promoter, with the *HES1* promoter analyzed as a positive control. Consistently, blockade of ICN1 generation by Compound E disrupted this interaction (Fig. 2e). Tentative NOTCH1 binding sites of *SHQ1* were constructed into a luciferase reporter vector; wild-type, but not mutant, DNA sequences are sufficient to activate luciferase expression when co- transfected with ICN1-expressing constructs (Fig. 2f). Our data provide compelling evidence that nuclear NOTCH1 specifically binds to the *SHQ1* promoter for direct transcriptional activation which may be the primary molecular mechanism underlying elevated SHQ1 expression in T-ALL.

### SHQ1 is required for T-ALL cell survival

**Fig. 3:**
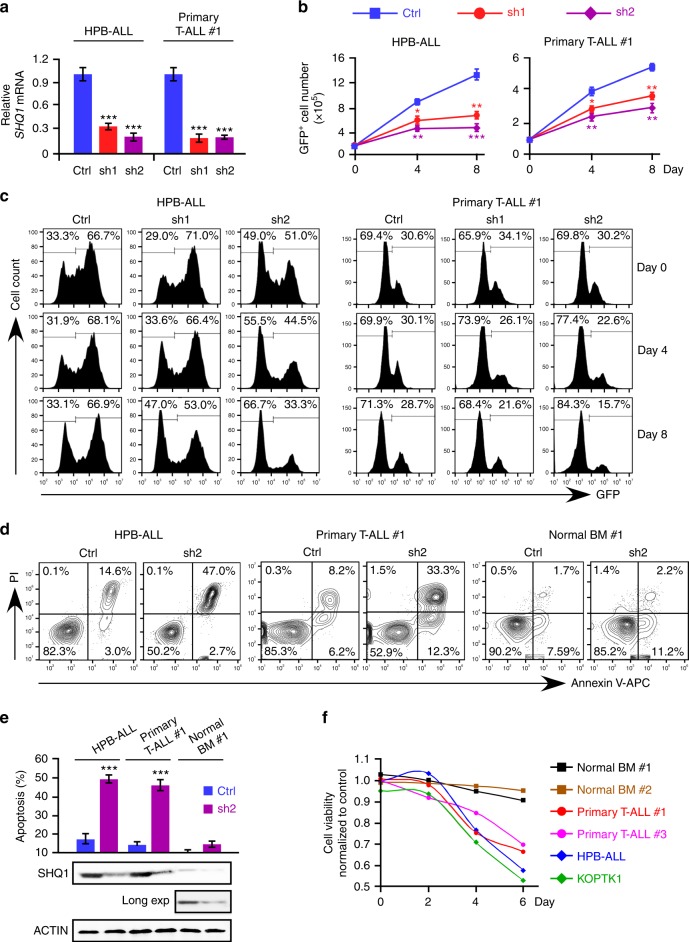
*SHQ1* depletion impairs T-ALL cell survival while it spares normal bone marrow cells. **a** HPB-ALL and patient-derived T-ALL (#1) cells were infected with lentiviruses expressing control (Ctrl, blue) or *SHQ1* shRNA (sh1-red or sh2-purple) with GFP as an expression marker. *SHQ1* mRNA levels were analyzed in GFP^+^ cells. **b** Live GFP^+^ cells were counted at the indicated time points and cell growth was plotted as shown. Cell counts of those expressing control shRNA, SHQ1 shRNA-1, or -2 are marked as blue square, red circle, or purple diamond. **c** Percentages of GFP^+^ cell populations were analyzed by flow cytometry at the indicated time points post infection. **d**, **e** Apoptotic cell death was analyzed by Annexin V-PI staining 6 days post infection and quantified in **e** (blue: control shRNA, purple: SHQ1 shRNA-2), along with immunoblots of SHQ1. Long exposure of SHQ1 bands in bone marrow cells is also shown (Long exp). **f** Cell viability was assessed by MTT assay in two T-ALL cell lines (HPB-ALL, blue and KOPTK1, green), two primary T-ALL cells (#1, red and #3, purple), and two normal human BM cells (#1, black and #2, brown) expressing either control or *SHQ1* shRNA. Absorbance readings of *SHQ1*-deficient cells relative to control cells were plotted as shown. Data shown represent the means (±SEM) of three biological replicates; *p < 0.05, **p < 0.01, ***p < 0.001, unpaired *t*-test (**a**, **b**, **e**)

To clarify the role of SHQ1 in T-ALL cell viability, we knocked it down in human T-ALL cells with two short hairpin RNAs (shRNAs) (Fig. 3a and Supplementary Fig. 3a). These shRNAs were cloned into modified pLKO.1 vector, in which the puromycin-resistant gene was substituted by the green fluorescent protein (GFP) gene^29^. As shown in Fig. 3b and Supplementary Fig. 3b, not only *SHQ1* deficiency in HPB-ALL and KOPTK1 cells but also in primary T-ALL resulted in marked cell growth inhibition. Consistently, *SHQ1*-depleted cells lost growth advantage as percentages of GFP^+^ populations dramatically declined over time, while cells expressing control shRNA remained unaffected (Fig. 3c and Supplementary Fig. 3c). Notably, *SHQ1* ablation induced noticeable apoptotic cell death in HPB-ALL, KOPTK1, and primary T-ALL cells, yet minimally affected normal bone marrow cells (Fig. 3d, e and Supplementary Fig. 4a-4b). In addition, we also analyzed SHQ1 function in normal murine thymus and murine T-ALL cells, and consistently found heightened cell death in leukemia cells upon *SHQ1* inactivation as compared to normal thymocytes (Supplementary Fig. 4c). Time course cell viability assay showed that *SHQ1* ablation resulted in poor cell survival in T-ALL but not in normal bone marrow, confirming an essential role of SHQ1 in the survival of transformed T cells (Fig. 3f). Cell cycle was not affected as a result of *SHQ1* deficiency (Supplementary Fig. 4d-4e), suggesting that *SHQ1* inactivation imposes a cytotoxic, rather than cytostatic, effect on T-ALL cells.

To evaluate the role of SHQ1 in different tumor types, we inactivated *SHQ1* in B-ALL (RS4;11), acute myeloid leukemia (AML) (HL-60), chronic myeloid leukemia (CML) (K562), and lung cancer (A549) cell lines. B-ALL, AML, and CML cells were all sensitive to *SHQ1* inactivation, whereas lung cancer cells were not (Supplementary Fig. 5a). In line with this observation, high *SHQ1* expression was associated with poor prognosis of T-cell lymphoma and AML, while it predicted favorable outcome in lung cancer (Supplementary Fig. 5b). Based on these data from various tumor types, we reason that SHQ1 may exert variable or even opposing roles in different tumor contexts.

### *SHQ1* depletion impedes T-cell leukemogenesis in vivo

**Fig. 4:**
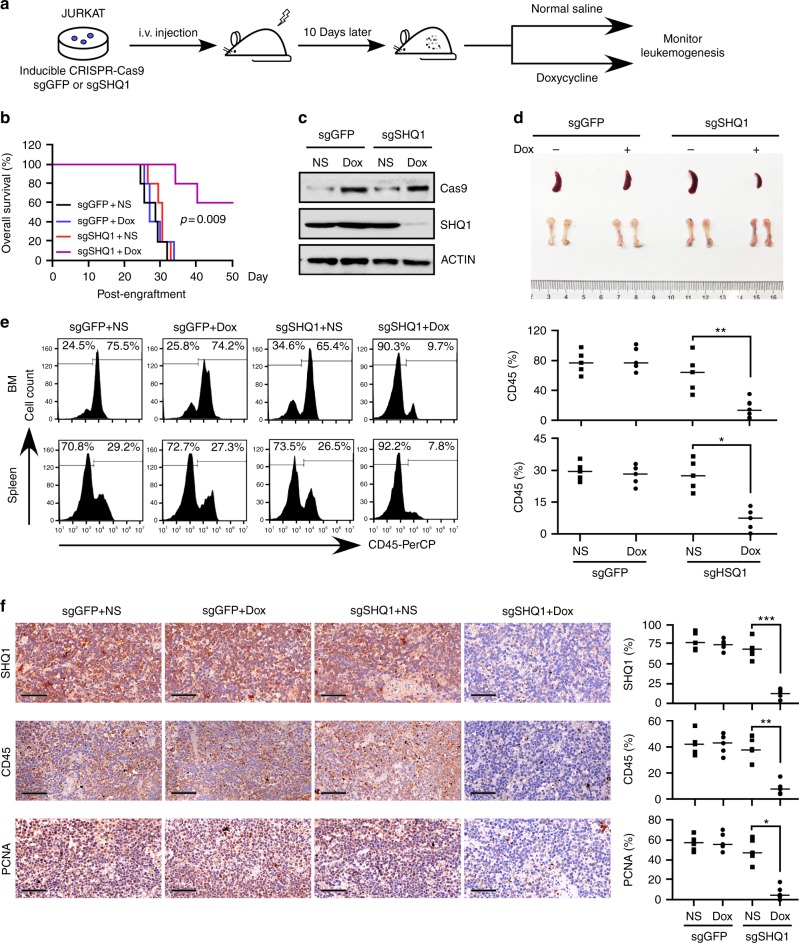
*SHQ1* knockout significantly decreases leukemia burden in the human T-ALL xenograft. **a** Schematic representation of human T-ALL xenograft. Two million JURKAT cells, expressing inducible CRISPR-Cas9 and sgRNA targeting GFP or SHQ1, were injected into irradiated NPG mice (1 gray). At 10 days post engraftment, doxycycline or normal saline was introduced into each allocated group for consecutive 7 days. **b** Kaplan–Meier survival curves of JURKAT xenografts in each group as indicated (*n* = 5 for each group). Survival of doxycycline-treated or untreated sgSHQ1 mice (purple and red lines) are compared with *p* value provided as shown (log-rank test). Between 28 and 35 days post engraftment, treated (blue line) or untreated (black line) sgGFP mice became moribund and 5 mice were taken out in each group and killed for assessment of leukemia burden. **c** Cas9 and SHQ1 were analyzed in human CD45^+^ bone marrow cells obtained from representative mice in each group. **d** Representative spleen and bone images of mice carrying JURKAT cells with or without *SHQ1* expression. **e** Human CD45^+^ cells from spleen and bone marrow (as shown in **d**) were analyzed by flow cytometry. Data from five individual mice were plotted and shown on the right. **f** Representative immunohistological images of SHQ1, human CD45, and PCNA in spleen sections from indicated mice. Scale bar, 50 μm. Histological stain was quantified using ImageJ; *p < 0.05, **p < 0.01, ***p < 0.001, unpaired *t*-test (**e**, **f**)

To assess the role of SHQ1 in leukemogenesis, we established a doxycycline-inducible *SHQ1* knockout JURKAT cell line using CRISPR/Cas9 system and transplanted these cells into immuno-compromised NPG mice, using non-specific single-guide RNA (sgRNA) targeting GFP as a control. Prior to transplantation, inducible *SHQ1* deletion was confirmed to suppress JURKAT cell growth in vitro (Supplementary Fig. 6). At 10 days post engraftment, animals were randomly divided into two groups. Doxycycline (200 mg kg^−1^) was administrated for consecutive 7 days to induce Cas9 expression and sgRNA-mediated cleavage (Fig. 4a). Mice bearing sgGFP-expressing JURKAT cells succumbed to T-ALL within 35 days, regardless of doxycycline treatment or not. In contrast, doxycycline treatment significantly improved animal survival rates and prolonged life span of mice transplanted with sgSHQ1-expressing JURKAT cells (Fig. 4b), associated with efficient *SHQ1* depletion (Fig. 4c). Consistently, mice bearing *SHQ1*-deficient cells manifested ameliorated splenomegaly and more reddish bones (Fig. 4d), as well as suppressed CD45^+^ leukemia cell dissemination in the BM and spleen (Fig. 4e, f). Immunohistochemical (IHC) staining confirmed that SHQ1 deficiency significantly decreased human CD45^+^ leukemia burden and cell proliferation (reflected by proliferating cell nuclear antigen (PCNA) staining) in the spleen (Fig. 4f). Similar results were obtained when we examined another HPB-ALL xenografts in which shRNA-mediated *SHQ1* silencing also decreased T-ALL burden in vivo (Supplementary Fig. 7).

**Fig. 5:**
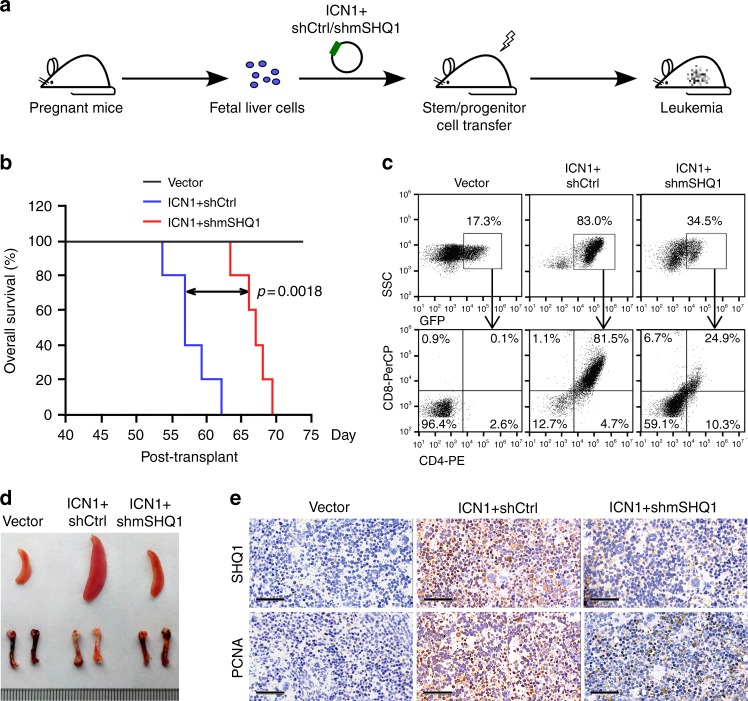
*SHQ1* depletion impedes leukemogenesis in NOTCH1-induced T-ALL model. **a** Graphical illustration of NOTCH1-induced T-ALL mouse model. Fetal liver cells were infected with MSCV retrovirus, enabling simultaneously expression of ICN1 and shRNA targeting non-specific control (shCtrl) or murine SHQ1 (shmSHQ1). Two million GFP^+^ transduced cells were intravenously injected into each irradiated recipient, followed by assessment of leukemia cell dissemination. **b** Kaplan–Meier survival curves of fetal liver cell transplants expressing vector (black line, *n* = 5), ICN1/shCtrl (blue line, *n* = 5), or ICN1/shmSHQ1 (red line, *n* = 5). The *p* value was determined by log-rank test. Around day 55 post transplantation, ICN1/shCtrl mice became moribund, then three mice in each group were killed for assessment of T-ALL in vivo. **c** Representative flow cytometry analysis of GFP^+^ and CD4^+^CD8^+^ leukemia cell distribution in each group. **d** Representative spleen and bone images in each transplant. **e** Representative histological images of murine SHQ1 and PCNA in spleen sections from each group. Scale bar, 50 μm

We next evaluated the role of SHQ1 in NOTCH1-induced T-ALL using retroviral ICN1 transduction and fetal liver cell transplant model. The retroviral vector MSCV-IRES-GFP^30^ allows co-expression of ICN1 and shRNA-of-interest in one construct. Retroviruses expressing empty vector, ICN1/control shRNA or ICN1/murine SHQ1 shRNA, with GFP as an expression marker, were transduced into hematopoietic stem/progenitor cells (HSPCs) from fetal livers of donor mice^31,32^. We then transplanted these HSPCs into irradiated recipients and assessed the onset of frank leukemia among these mice (Fig. 5a). Mice expressing ICN1/control shRNA succumbed to T-ALL in about 2 months, whereas most mice expressing ICN1/murine *SHQ1* shRNA remained alive when ICN1/control shRNA mice became moribund (Fig. 5b). Compared to the in vivo expansion of HSPCs transduced with ICN1/control shRNA, *SHQ1* depletion significantly decreased accumulation of GFP^+^ cells (34.5% vs 83%), in which neoplastic CD4^+^CD8^+^ lymphoblasts were vastly reduced (24.9% vs 81.5%) (Fig. 5c), resulting in more reddish bones and much smaller spleen size (Fig. 5d). Again, PCNA staining manifested decreased cell proliferation in the spleen associated with diminished SHQ1 expression as a consequence of shRNA-mediated gene silencing (Fig. 5e). Notably, *SHQ1* depletion had minimal effect on HSPC homing efficiency (Supplementary Fig. 8), ruling out the possibility that the phenotype of delayed leukemogenesis was due to defective homing or engraftment of HSPCs. Therefore, results from two complementary murine T-ALL models demonstrate that SHQ1 plays a vital role in T-ALL initiation and progression.

### *SHQ1* depletion affects pre-mRNA splicing

**Fig. 6:**
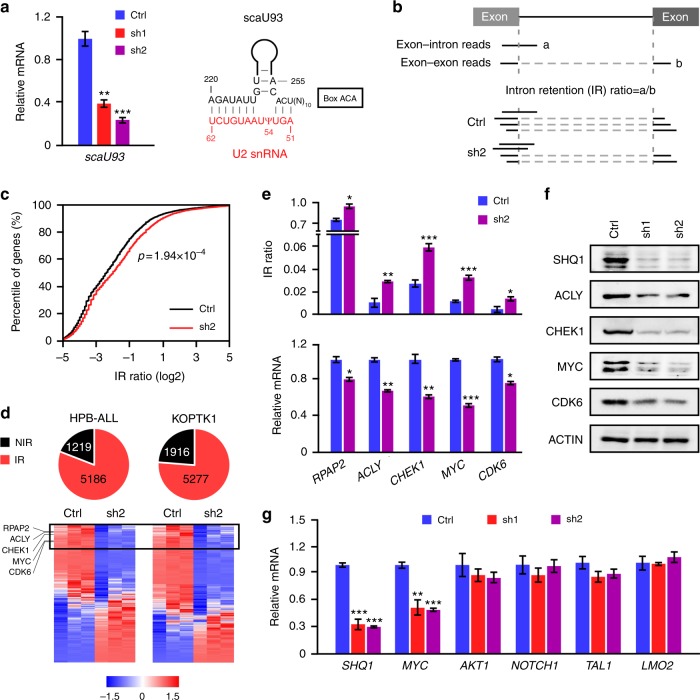
*SHQ1* depletion affects pre-mRNA splicing efficiency. HPB-ALL or KOPTK1 cells were infected with lentiviruses expressing control shRNA (Ctrl) or *SHQ1* shRNA (sh1 or sh2). **a** H/ACA snoRNA scaU93, base paired with U2 snRNA and guiding its pseudouridylation, in HPB-ALL cells expressing control shRNA (blue), SHQ1 shRNA-1(red), or shRNA-2(purple) was analyzed by qPCR. **b** Schematic of intron retention (IR) analysis. Exon–intron reads (a) relative to exon–exon reads (b) are defined as IR ratio which reflects the magnitude of pre-mRNA accumulation. **c** Empirical cumulative distribution of IR coefficients for 6809 genes in HPB-ALL cells. Curves represent IR ratio in control (black) and shRNA-2 mediated *SHQ1* depletion (red). A rightward shift in the curve representing *SHQ1*-depleted cells indicates increased IR, namely increased pre-mRNA accumulation upon *SHQ1* knockdown. **d** Genome-wide splicing and gene expression were analyzed in HPB-ALL and KOPTK1 cells. Numbers of genes with altered IR ratio were plotted in red at the pie chart, and those with no intron retention (NIR) were marked in black. Genes with IR ratios (*SHQ1* knockdown vs control) more than 1.15 are considered aberrant intron retention. Heatmaps of mature mRNA expression from intron-retained genes are presented at the bottom (red indicates increased and blue decreased). Top genes with most decreased mature mRNA associated with increased IR ratios in *SHQ1*-deficient cells are highlighted in the box. **e**, **f** Validation of candidate genes shown in the box from **d** by assessing IR ratio, mature mRNA (**e**), and protein expression (**f**) in HPB-ALL cells. **g** Analysis of T-ALL associated oncogene expression in HPB-ALL cells by qPCR. In **e** and **g**, blue, red, or purple bars represent indicated RNA quantifications in control shRNA, *SHQ1* shRNA-1, or -2-expressing cells. Data shown represent the means (±SEM) of three biological replicates; *p < 0.05, **p < 0.01, ***p < 0.001, unpaired *t-*test (**a**, **e**, **g**) or Kolmogorov–Smirnov test (**c**)

As a crucial factor for the assembly of H/ACA snoRNPs^19^, *SHQ1* loss often leads to degradation of the associated snoRNAs^18^. We confirmed the diminished expression of scaU93, an H/ACA snoRNA responsible for U2 snRNA pseudouridylation at the 54 site^33^, in *SHQ1*-depleted HPB-ALL cells (Fig. 6a). As expected, an in vitro *N*-cyclohexyl-*N*-(2-morpholinoethyl)-carbodiimid-methop-toluolsulfonate (CMC)-primer extension pseudouridylation assay^34^ manifested less U2 snRNA pseudouridylation in *SHQ1*-deficient cells (Supplementary Fig. 9). We reasoned that inefficient U2 snRNA modification would impair mRNA splicing. RNA-Seq was then performed in HPB-ALL and KOPTK1 cells expressing *SHQ1* or control shRNA to assess pre-mRNA splicing. Splicing analysis was restricted to reads directly spanning exon–intron or exon–exon junction sequences (HPB-ALL, 61,456 junctions in 6809 genes; KOPTK1, 78,976 junctions in 7905 genes). Reads spanning exon–intron junctions are considered as unspliced form (a) and reads across exon–exon junctions as spliced form (b). Intron retention (IR) ratio (a/b) reflects the magnitude of pre-mRNA accumulation (Fig. 6b). As predicted, *SHQ1* depletion caused widespread intron retention reflected by increased IR ratios (Fig. 6c). Indeed, 81% of genes in HPB-ALL and 73% of genes in KOPTK1 cells exhibited differential intron retention upon *SHQ1* inactivation (Fig. 6d). Intron-retaining pre-mRNAs often fail to complete mRNA maturation and are commonly degraded via quality control mechanisms^35^. We then focused on the most downregulated genes with splicing alteration in *SHQ1* loss and selected five candidates, *RNA polymerase II-associated protein 2* (*RPAP2*), *ATP citrate lyase* (*ACLY*), *checkpoint kinase 1* (*CHEK1*), *MYC*, and *cyclin-dependent kinase 6* (*CDK6*) (Fig. 6d). Their increased intron retentions were validated in *SHQ1-*depleted HPB-ALL (Fig. 6e) and primary T-ALL cells (Supplementary Fig. 10), consequently leading to decreased mature mRNA and protein levels (Fig. 6e, f). As a major proto-oncogene in T-ALL^10,36^, *MYC* gained our attention. To test whether SHQ1 globally affects T-ALL-associated oncogenes or specifically regulates *MYC*, we examined *MYC*, *AKT1*, *NOTCH1*, *TAL1*, and *LMO2* expression in response to *SHQ1* loss. Only *MYC* expression was significantly decreased (Fig. 6g), which is consistent with the findings from RNA-Seq (Fig. 6d and data not shown). We thus identify *MYC* as one of the most prominent genes downstream of the NOTCH1–SHQ1 axis.

### SHQ1 modulates *MYC* gene splicing and expression

**Fig. 7:**
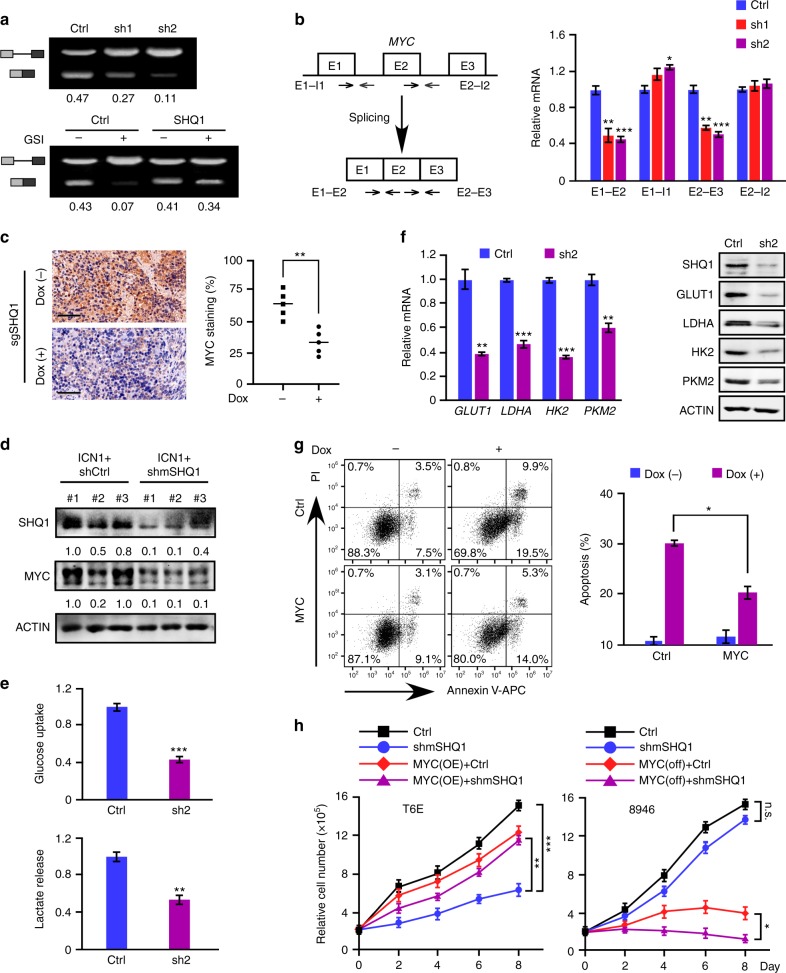
SHQ1 regulation of *MYC* gene splicing and expression is important for T-ALL cell survival. **a** *MYC* minigene splicing was analyzed in 293T cells expressing control (Ctrl) or *SHQ1* shRNA (sh1 or sh2) (top), and also in 293T cells expressing SHQ1 or empty vector in the presence of DMSO or GSI (Compound E, 1 μM) (bottom). Semi-quantitative RT-PCR was performed to analyze unspliced and spliced RNA. Splicing efficiency was calculated and shown as the ratio of spliced verse total RNA. **b** Schematic of *MYC* gene with three exons and two introns. Specific primer sets were designed to amplify pre-mRNA (E1–I1 and E2–I2) and mature mRNA (E1–E2 and E2–E3) (left). Endogenous *MYC* pre-mRNA and mature mRNA in HPB-ALL were analyzed by qPCR (right). Blue, red, or purple bars represent indicated RNA quantifications in control shRNA, *SHQ1* shRNA-1, or -2-expressing cells. **c** Representative immunohistological images of MYC in spleen sections from mice bearing sgSHQ1-JURKAT cells injected with or without doxycycline (Fig. 4d). Scale bar, 50 μm. Histological stain was quantified using ImageJ and plotted on the right. **d** Immunoblots of SHQ1 and MYC in GFP^+^ cells from 3 fetal liver HSPC transplant samples in Fig. 5d. **e** HPB-ALL cells were infected with lentiviruses expressing control (Ctrl, blue) or *SHQ1* shRNA (sh2, purple). Glucose uptake and lactate secretion were examined 4 days post infection, normalized to the same number of live cells. **f** Analysis of MYC target genes implicated in glycolysis by qPCR and immunoblots in control or *SHQ1-*deficient HPB-ALL cells 4 days post infection. Blue or purple bars represent indicated RNA quantifications in control shRNA, *SHQ1* shRNA-2-expressing cells. **g** *SHQ1* inducible-knockout JURKAT cells were overexpressed with *MYC* or blank control. Cell death was analyzed and quantified with (purple) or without (blue) doxycycline (Dox, 1 μg ml^−1^). **h** Cell growth was assessed using murine T-ALL T6E and 8946 cells. T6E cells were infected with MSCV-shControl (Ctrl, black), MSCV-shmSHQ1 (shmSHQ1, blue), MSCV-shControl-MYC (MYC OE+Ctrl, red), or MSCV-shmSHQ1-MYC (MYC OE+shmSHQ1, purple) as denoted. Sorted GFP^+^ cells were used for cell growth analysis (left). In all, 8946 cells were infected with MSCV-shControl (Ctrl) or MSCV-shmSHQ1 (shmSHQ1). Sorted GFP^+^ cells were subjected to doxycycline treatment (1 μg ml^−1^) to suppress human MYC transgene expression (MYC off+Ctrl, red; MYC off+shmSHQ1, purple), with PBS treatment as a control (Ctrl, black; shmSHQ1, blue), followed by cell growth analysis (right). Data shown represent the means (±SEM) of three biological replicates; *p < 0.05, **p < 0.01, ***p < 0.001; n.s. non-significant, unpaired *t-*test (**b**, **c**, **e**, **f**, **g**, **h**)

To further analyze *MYC* pre-mRNA splicing, we expressed a minigene carrying *MYC* intron 2 and flanking exons in 293T cells (Supplementary Fig. 11). *SHQ1* knockdown led to noticeable accumulation of unspliced RNA, resulting in less mature RNA. We observed a more profound defect on minigene splicing upon pharmacological inhibition of NOTCH which was significantly reversed by exogenous expression of *SHQ1* (Fig. 7a). Endogenous *MYC* in HPB-ALL cells was also assessed with primers designed to amplify pre-mRNA (E1–I1 and E2–I2) and mature mRNA (E1–E2 and E2–E3). As shown in Fig. 7b, mature *MYC* mRNA, but not pre-mRNA, was significantly reduced in *SHQ1* knockdown cells. Again, deregulation of endogenous *MYC* splicing upon NOTCH inhibition was efficiently rescued by SHQ1 overexpression (Supplementary Fig. 12). More importantly, diminished MYC expression upon *SHQ1* ablation in vivo was also confirmed in JURKAT cell xenograft (Fig. 7c) and fetal liver cell transplant (Fig. 7d).

We next analyzed MYC-regulated aerobic glycolysis upon *SHQ1* deficiency and found significantly impaired glucose uptake and lactate secretion in *SHQ1*-depleted HPB-ALL and KOPTK1 cells (Fig. 7e and Supplementary Fig. 13a), associated with downregulation of MYC target genes implicated in glycolysis (Fig. 7f and Supplementary Fig. 13b). Notably, these glycolysis defects were efficiently rescued by enforced *MYC* expression (Supplementary Fig. 13c). To evaluate the role of SHQ1-regulated MYC in cell viability, we overexpressed MYC in JURKAT cells, in which doxycycline treatment induced *SHQ1* knockout (Supplementary Fig. 6). *MYC*-overexpressed cells significantly ameliorated cell apoptosis resulting from *SHQ1* depletion (Fig. 7g). Enforced *MYC* expression also rescued cell death caused by SHQ1 inactivation in HPB-ALL and KOPTK1 cells (Supplementary Fig. 13d) as well as murine T-ALL T6E cells (Fig. 7h). Conversely, murine T-ALL 8946 cells constitutively expressing a human *MYC* transgene^10^, which does not require RNA splicing for expression, were more refractory to *SHQ1* depletion. When doxycycline treatment switched off *MYC* expression, *SHQ1* depletion induced more severe growth inhibition, presumably due to suppression of endogenous murine Myc expression (Fig. 7h). Collectively, these results provide strong evidence supporting that SHQ1 serves an important role in modulating *MYC* splicing and MYC acts as a crucial downstream effector mediating the function of SHQ1 in T-ALL cell survival.

## Discussion

**Fig. 8:**
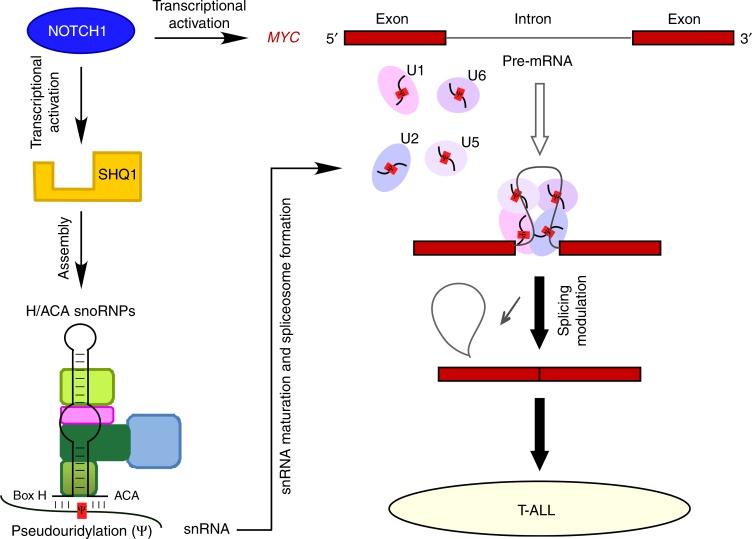
Model depicting the NOTCH1–SHQ1–MYC axis in promoting T-cell leukemogenesis. NOTCH1 directly activates *SHQ1* transcription. The resulting accumulation of SHQ1 protein optimizes the assembly of H/ACA snoRNPs and snRNA pseudouridylation, potentiating *MYC* pre-mRNA splicing. In addition to NOTCH1 transcriptional regulation of *MYC*, NOTCH1 induces *SHQ1* expression and enhances *MYC* RNA processing, ultimately maximizing MYC expression in T-ALL. See discussion for more details

Based on gene expression profiling analysis, we identify that SHQ1, whose expression is aberrantly upregulated in T-ALL, contributes to leukemogenesis. We demonstrate SHQ1, directly driven by NOTCH1, is required for T-ALL cell survival in vitro and expansion in vivo. This important role of SHQ1 attributes to the assembly of H/ACA snoRNPs that mediates snRNA pseudouridylation and mRNA splicing. We here provide mechanistic evidence demonstrating regulation of *MYC* splicing by SHQ1 as an important event involved in T-cell leukemogenesis (Fig. 8), thus revealing the tumor-supporting role of SHQ1 in human cancers that links SHQ1 to RNA splicing and leukemogenesis.

Regulatory mechanism and functional role of SHQ1 in human cancers are largely unknown. Using T-ALL as a model system, we reveal a molecular mechanism underlying SHQ1 expression in human cancers. NOTCH1 transcriptional complex directly binds to the promoter region of *SHQ1* and activates its transcription. Given aberrant, sustaining NOTCH1 activation in T-ALL, this regulatory mechanism may explain common elevation of *SHQ1* expression in various T-ALL subtypes. Consistently, previous publications suggest that *SHQ1* expression is dependent on NOTCH1 activity in T-ALL^11,26^. Being an important effector downstream of NOTCH1 signaling, enhanced SHQ1 expression is essential for T-ALL cell survival. Similarly, SHQ1 plays an essential role in B-ALL, AML, and CML cells, and high *SHQ1* expression correlates with poor prognosis in T-cell lymphoma and AML. However, *SHQ1* deficiency in lung cancer cell line A549 induces minimal cell death and higher expression of *SHQ1* is associated with better overall survival in lung cancer patients. These findings are agreeable to prior reports that non-small cell lung cancer^37^ and prostate cancer^38–41^ exhibit aberrantly low expression or genomic deletion of *SHQ1*. Based on these data from various tumor types, we reason the supporting or suppressing role of SHQ1 in human cancers is highly context dependent probably through regulating splicing of distinct downstream genes.

Our in-depth mechanistic analysis reveals that SHQ1 modulates *MYC* splicing and expression. In line with prior reports that NOTCH1 directly activates *MYC* transcription through exquisite mechanisms^27,36,42^, these findings add an extra layer of complexity on MYC regulation by SHQ1, H/ACA snoRNPs, and spliceosomes. When exploring the molecular mechanism underlying specific regulation of *MYC* splicing by SHQ1, we identified a putative branch site (5′-UCCUG (A) C-3′) within *MYC* intron 2. The nucleotide base paired with pseudouridine (Ψ) in U2 snRNA is guanine (G) or cytosine (C) instead of the common nucleotide adenosine (A) (Supplementary Fig. 14a). Generally the presence of Ψ enables more efficient association between U2 snRNA and pre-mRNA substrate^13^. In this case, splicing of *MYC* intron may be more dependent on Ψ because this modification improves the capacity and flexibility of unconventional base pair with G or C. As predicted, mutagenesis of G/C to A in the *MYC* minigene significantly ameliorated the splicing defect resulting from *SHQ1* inactivation (Supplementary Fig. 14b), presumably due to a more stable U–A pair to initiate splicing process to some extent. It is notable that similar *cis*-elements are found in other candidate genes whose splicing and expression are markedly affected by *SHQ1* loss (Supplementary Fig. 14c), suggesting a potential, common mechanism involved in SHQ1-regulated splicing.

Additional effectors downstream of SHQ1 may contribute to T-ALL as well. Indeed, our screen identifies CDK6 as a SHQ1 responder and their expression correlates to each other in primary T-ALLs (Supplementary Fig. 15). Inhibition of CDK6 leads to remarkable anti-leukemia effect in T-ALL cell lines as well as mouse model^43,44^. Modulation of other T-ALL-associated pro-oncogenic genes suggests that SHQ1 may coordinate a leukemogenic program enabling T-cell transformation. SHQ1 is also responsible for telomerase RNP assembly and important for telomere maintenance^19^, yet its function in T-ALL seems to be telomere independent. Expression of telomerase H/ACA RNA (hTR) and reconstitution of telomerase activity failed to rescue T-ALL cells from *SHQ1* depletion (Supplementary Fig. 16). Similar findings were reported in neuroblastoma that telomere reconstitution was unable to restore H/ACA snoRNP loss of function^45^.

Taken together, our data suggest that T-ALL may have an increased dependency on SHQ1-mediated snRNA pseudouridylation and functionally intact spliceosome. Oncogene activation has been shown to increase total RNA and protein production in various tumor contexts. While such increases in RNA and protein production may endow cancer cells with pro-tumorigenic hallmarks, this increase in synthesis may also generate heightened burden on cancer cells to process these macromolecules properly^46^. Our findings provide an example that, in order to adapt the oncogenic stress, NOTCH1 activates the expression of SHQ1 which optimizes snRNA modification and maximizes spliceosomal potential to accommodate greater volumes of total RNA. As such, partial modulation or inhibition of spliceosome may be detrimental to T-ALL cells. Hence, our findings highlight the indispensable role of SHQ1 for optimal spliceosome function in T-ALL which may offer therapeutic opportunity for T-ALL patients by targeting of SHQ1 or spliceosome.

## Methods

### Cell culture

The 293T, JURKAT, and CCRF-CEM cells were purchased from American Type Culture Collection (ATCC). Human T-ALL cell lines SIL-ALL, HPB-ALL, KOPTK1, CUTLL1, and DND41, and murine T-ALL cell lines T6E, G4A2, and 8946 were kindly provided by Dr Warren Pear (University of Pennsylvania). T-ALL cell lines were grown in complete RPMI-1640 (Hyclone) supplemented with 10% fetal bovine serum (FBS, Hyclone), 1% penicillin/streptomycin (Hyclone), 1% non-essential amino acids (Gibco), 2 mM l-glutamine (Sigma), 1 mM sodium pyruvate (Sigma), and 100 μM β-mercaptoethanol (Sigma). Primary T-ALL cells were co-cultured with MS5-DL1 feeder cells in WIT-L medium supplemented with stem cell factor (SCF) (50 ng ml^−1^), interleukin (IL)-2 (10 ng ml^−1^), IL-7 (10 ng ml^−1^), and insulin-like growth factor-1 (10 ng ml^−1^)^47^. Human normal bone marrow cells were maintained in minimum essential medium-α supplemented with 20% FBS and granulocyte-colony stimulating factor (50 ng ml^−1^). The 293T cell was maintained in Dulbecco's modified Eagle's medium (DMEM; Hyclone) containing 10% FBS and 1% penicillin/streptomycin (Hyclone). All cell lines were authenticated using the variable number of tandem repeats PCR assay, cultured for fewer than 6 months after resuscitation, and tested for mycoplasma contamination every 3 months using MycoAlert (Lonza)^48^. Human primary specimens were obtained with informed consents from Center for Medical Genetics, Ghent University, Ghent, Belgium; Center for Cancer Research, Boston University School of Medicine, Boston, USA; Guangzhou Institutes of Biomedicine and Health, Chinese Academy of Sciences, Guangzhou, China; Union Hospital and Tongji Hospital, Wuhan, China.

### RNA extraction and quantitative real-time PCR

Total cellular RNA was extracted using TRIzol (Invitrogen) and random primed RNAs (1 μg) were reverse transcribed with RevertAid first-strand complementary DNA synthesis kit according to the manufacturer’s instructions (Thermo Scientific). Quantitative PCR was conducted using FAST SYBR Green Master Mix on CFX Connect Real-Time PCR System (Bio-Rad). Relative expression of the mRNA was calculated by 2^−ΔΔCt^ method and normalized to *ACTIN*. Specific PCR primer sequences are listed in Supplementary Table 1.

### Immunoblotting

Cells were lysed with RIPA buffer (50 mM Tris-HCl pH 7.4, 150 mM NaCl, 1% Triton X-100, 1% sodium deoxycholate, 0.1% SDS, 2 mM sodium pyrophosphate, 25 mM β-glycerophosphate, 1 mM EDTA, 1 mM Na_3_VO_4_, and 0.5 μg ml^−1^ leupeptin), and protein concentrations determined using Bradford reagent (Bio-Rad). 30–50 μg total cellular proteins were then subjected to SDS-polyacrylamide gel and transferred to polyvinylidene difluoride membrane (Bio-Rad). After being blocked with 5% fat free milk, blots were generally incubated with primary antibodies at 4 °C overnight. Appropriate horseradish peroxidase-conjugated secondary antibodies were applied for 1–2 h at room temperature before detection with SuperSignal Chemiluminescent Substrate (Bio-Rad). Densitometric analyses of protein abundance were determined by ImageJ software. All uncropped blotting images are presented in Supplementary Fig. 17 and 18. Antibodies used in the experiments include β-ACTIN (1:2000, A5441, Sigma-Aldrich), SHQ1 (1:500, ab110692, Abcam), MYC (1:1000, sc-764, Santa Cruz), TAL1 (1:1000, sc-393287, Santa Cruz), CHEK1 (1:1000, sc-8408, Santa Cruz), CDK6 (1:1000, A1545, ABclonal), GATA3 (1:1000, A1638, ABclonal), ACLY (1:1000, #4332, Cell Signaling Technology), cleaved NOTCH1 (1:1000, #4147, Cell Signaling Technology), HK2 (1:1000, #2106, Cell Signaling Technology), LDHA (1:1000, #2012, Cell Signaling Technology), GLUT1 (1:1000, ab652, Abcam) and PKM2 (1:1000, ab38237, Abcam).

### ChIP assay

ChIP was performed using human T-ALL SIL-ALL cells^10,49^. These cells were treated with dimethyl sulfoxide (DMSO) or Compound E (1 μM, Merck) for 24 h, then fixed with 1% paraformaldehyde at room temperature for 10 min. Cells were subjected to a Bioruptor Pico Sonifier to shear chromatin DNA to a size range of 500–1000 bp. Precleared chromatin was immunoprecipitated with antiserum against intracellular NOTCH1 or rabbit IgG (sc-3888, Santa Cruz Biotechnology) for 16 h at 4 °C. Antibody–chromatin complexes were pulled down with protein G agarose/salmon sperm DNA beads (Roche) (1 h, 4 °C). The eluted material was reverse-cross-linked and treated with proteinase K (40 μg ml^−1^). Immunoprecipitated DNA was purified by phenol/chloroform extraction, eluted by distilled H_2_O, and quantified by CFX Connect Real-Time PCR System (Bio-Rad) using specific primers listed in Supplementary Table 1.

### Luciferase reporter assay

The wild-type or mutant *SHQ1* promoter sequences were amplified using specific primers listed in Supplementary Table 1. The resulting DNA fragments were constructed into pGL3-basic firefly luciferase reporter vector (Promega). To detect luciferase reporter activity, 0.8 μg pGL3 expressing the *SHQ1* promoter (or empty vector) and 0.2 μg pcDNA3-ICN1 plasmid, along with 50 ng pRL-TK *Renilla* luciferase reporter construct, were co-transfected into 293T cells using Lipofectamine 2000 (Thermo Fisher Scientific). Luciferase activities were measured 24 h later using Dual Luciferase Reporter Assay System (Promega)^49^. Firefly luciferase activities were normalized with *Renilla* luciferase control values, and relative to values from the empty vector lysate.

### Lentiviral or retroviral transduction

For viral production, lentiviral vectors pLKO.1 or pCDH were used for plasmid construction and transfected into 293T cells simultaneously with helper plasmids (pMD2.G and psPAX2); retroviral vectors MSCV-IRES-GFP or MigR1 were used for plasmid construction and transfected into 293T cells simultaneously with packaging plasmids (pCgp and pHIT). Viral supernatants were generally collected 16–24 h post transfection. Transduction of T-ALL cells was carried out as follows^49^. 1 × 10^6^ cells were incubated with 0.5 ml viral supernatant and 8 μg ml^−1^ polybrene (Sigma) in a final volume of 2 ml for 0.5 h, and subjected to centrifugation at 1000 × *g* for 90 min at room temperature. Cells were then supplemented with 3 ml fresh medium and continued culture for additional 48 h.

### Flow cytometry analysis

Cells with GFP fluorescence or stained with indicated antibodies were resuspended in phosphate-buffered saline (PBS). Acquisition was performed on an Accuri C6 (BD Biosciences) and live cells were gated based on FSC-A and SSC-A characteristics. Data were analyzed with FlowJo software (TreeStar). Flow cytometric sorting was conducted using a FACS Aria (BD Biosciences).

### Inducible SHQ1 knockout cell generation

DNA sequences encoding specific sgRNA were constructed into pLX-sgRNA vector according to the instruction (#50662, Addgene). Inducible Cas9 JURKAT cells were generated as described^50^. JURKAT cells were infected with lentivirus expressing pCW-Cas9 (#50661, Addgene), selected by puromycin (2 μg ml^−1^) for 48 h, and clonally sorted into 96-well tissue culture plates containing 200 μl of media. Upon doxycycline (1 μg ml^−1^) treatment, the single colony with the greatest fold change in Cas9 expression was selected for further pLX-sgGFP or pLX-sgSHQ1 lentiviral transduction. Positive colonies were subsequently screened out by blasticidin (10 μg ml^−1^) treatment for 72 h.

### Human T-ALL xenograft

JURKAT xenografts were carried out as previously described^48,49^. The 4–6-week-old female NPG mice (Beijing Vital River Laboratory Animal Technology Co., Ltd.) were irradiated at 1 gray before tail vein injection of 2 × 10^6^ JURKAT cells infected with inducible Cas9 and SHQ1 sgRNA (or GFP sgRNA). Weekly monitoring of mice for circulating leukemia cells in peripheral blood was performed by analysis of human CD45 expression with flow cytometry. At 10 days post engraftment, mice expressing each specific sgRNA were randomly divided into two groups, and subjected to intraperitoneal injection of doxycycline (200 mg kg^−1^) or normal saline for 7 days. Mice were killed when demonstrating characteristic disease symptoms or becoming moribund. Cells were then isolated from spleens by mechanical disaggregation and red blood cell lysis, and collected from bone marrow by flushing of femurs with PBS. The human CD45 surface marker was assessed by flow cytometry analysis of leukemia burden in vivo. Spleen sections were prepared for immunohistochemistry staining. This work was performed under animal ethical regulations and the study protocol was approved by the Institutional Animal Care and Use Committee of Wuhan University.

### Fetal liver cell transplantation

Fetal liver cell transplantation was performed as previously described^51^. Day 13.5–16.5 pregnant C57BL/6 mice (Beijing Vital River Laboratory Animal Technology Co., Ltd.) were killed to obtain fetal livers which were minced and grown at approximately 3 × 10^6^ cells ml^−1^ in conditions supporting hematopoietic stem cell growth. Cells were grown in 37% DMEM (Hyclone) and 37% Iscove's modified Dulbecco's medium (Hyclone) supplemented with 20% FBS (Hyclone), 2% l-glutamine (200 mM), 100 U ml^−1^ penicillin/streptomycin, 50 nM 2-mercaptoethanol, 4% 0.45 μm filtered WEHI-3B supernatant, 0.2 ng ml^−1^ recombinant murine IL-3, 2 ng ml^−1^ recombinant murine IL-6, and 20 ng ml^−1^ recombinant murine SCF (PeproTech). The retroviral vector used in this study was a modified MSCV-IRES-GFP vector^30^, kindly provided by Dr. Chong Chen from Sichuan University, China, which allows co-expression of ICN1 and shRNA targeting SHQ1 (or GFP). Retroviruses expressing MSCV-IRES-GFP (empty vector), MSCV-shControl-ICN1-IRES-GFP, or MSCV-shSHQ1-ICN1-IRES-GFP were transduced into fetal liver cells cultured in vitro. For bone marrow reconstitution experiments, 6–8-week-old C57BL/6 recipient mice were subjected to lethal irradiation (9 gray), and reconstituted 6 h later with a total of 2 × 10^6^ GFP^+^ infected fetal liver cells by tail vein injection. Mice received enrofloxacin-containing drinking water for 2 weeks post transplant. GFP^+^ or CD4^+^CD8^+^ cells in peripheral blood were subsequently analyzed by flow cytometry to trace the onset of leukemia. Mice were monitored for survival and killed when moribund or demonstrating obvious clinical distress. Single-cell suspensions from bone marrow and spleen were processed for flow cytometry and gene expression analysis. Spleen sections were prepared for immunohistochemistry staining. This work was performed under animal ethical regulations and the study protocol was approved by the Institutional Animal Care and Use Committee of Wuhan University.

### Immunohistochemistry

The IHC analysis was carried out using Histostain-Plus IHC Kit (Thermo Fisher Scientific)^49^. Paraffin-embedded tissue sections were incubated with the antibodies against CD45 (1:100, 13–9457, eBioscience), SHQ1 (1:100, ab110692, Abcam), c-Myc (1:200, sc-764, Santa Cruz), or PCNA (1:200, sc-56, Santa Cruz) overnight at 4 °C. These slides were then subjected to horseradish peroxidase-linked secondary antibodies for 1 h at room temperature. Staining was visualized by the DAB substrate kit (Vector Labs) and representative areas of each stained tissue section were imaged at ×400 magnification. ImageJ software was used to quantify the staining results.

### RNA-seq

HPB-ALL or KOPTK1 cells were infected with pLKO.1-shRNA against SHQ1 or GFP control lentiviruses and cultured for 48 h, followed by puromycin (2 μg ml^−1^) selection for additional 48 h. Total RNA was isolated using Trizol reagent (Thermo Fisher Scientific). RNA samples were rRNA depleted, and RNA libraries were constructed using TruSeq RNA Library Prep Kit v2 (Illumina) and sequenced as 150 bp paired-end reads by Illumina HiSeq 2000 (Beijing Annoroad Co. Ltd). RNA-seq NGS reads quality was evaluated using FastQC. Efficiency of RNA splicing was analyzed as previously described^46^. Briefly, reads were mapped to the human genome reference assembly (hg19) using Bowtie 2. The ratio of exon–intron reads to exon–exon reads was calculated as junction IR. Mature mRNA expression was determined by normalized RPKM values (Reads Per Kilobases per Million reads) of aligned exon–exon transcripts. We restricted the above-mentioned analyses to high confidence genes with an average of at least 10 total junction reads and RPKM ≥100 in the control samples. The heatmap was generated using Cluster 3.0 and TreeView software.

### Minigene assay

Human *MYC* intron 2 and about 500 bp of flanking exonic sequences were amplified by PCR from genomic DNA and cloned into a hybrid minigene pBluescript KS construct (a generous gift from Dr. Hai-Ning Du, Wuhan University). pLKO.1-SHQ1 shRNA or pCDH-SHQ1 plasmid (1 μg) were individually transfected into 293T cells using Lipofectamine 2000 (Thermo Fisher Scientific) in a final volume of 1 ml. After 24 h, the MYC minigene was transfected into 293T cells prior to additional 24 h of cell culture. Cells were then harvested and RNA was isolated, reverse transcribed, and subjected to PCR analysis (30 s at 94 °C, 30 s at 57 °C, 60 s at 72 °C; 30 cycles)^52^. The resulting PCR products were separated on a 1% agarose gel. Unspliced and spliced minigene bands were quantified using ImageJ and values were calculated as ratios of spliced/total RNA products. The PCR primers used are shown in Supplementary Table 1.

### Glucose uptake and lactate secretion assays

Glucose uptake and lactate secretion analysis were carried out using respective colorimetric assay kits according to the manufacture’s instruction (BioVision). Briefly, 4 × 10^5^ cells were inoculated in each well of 6-well plates. After 48 h of incubation, cell culture media were collected to quantify glucose and lactate. Consumed glucose and released lactate were calculated and normalized to the same live cell numbers.

### Statistical analysis

Log-rank analysis was used to evaluate differences in Kaplan–Meier survival curves. Kolmogorov–Smirnov test was applied to analyze empirical cumulative distributions of IR scores in splicing analysis. Other statistical analysis was calculated using two-tailed Student’s *t*-test, with *p* < 0.05 considered significant.

## Electronic supplementary material

Supplementary Information

Description of Additional Supplementary Files

Supplementary Data 1

## Data Availability

RNA-Seq data that support the findings of this study have been deposited in Gene Expression Omnibus with the accession code GSE117264. All other relevant data are available from the corresponding authors.
