# Supplementary material for: SHQ1 regulation of RNA splicing is required for T-lymphoblastic leukemia cell survival
Source: Nat Commun. 2018 Oct 15;9:4281. doi: 10.1038/s41467-018-06523-4 (PMC6189109; doi:10.1038/s41467-018-06523-4)
Supplement: Supplementary file 1 — Supplementary Information [file 41467_2018_6523_MOESM1_ESM.pdf]

Supplymentary Information

SHQ1 regulation of RNA splicing is required for T-lymphoblastic leukemia cell survival

Su et al.

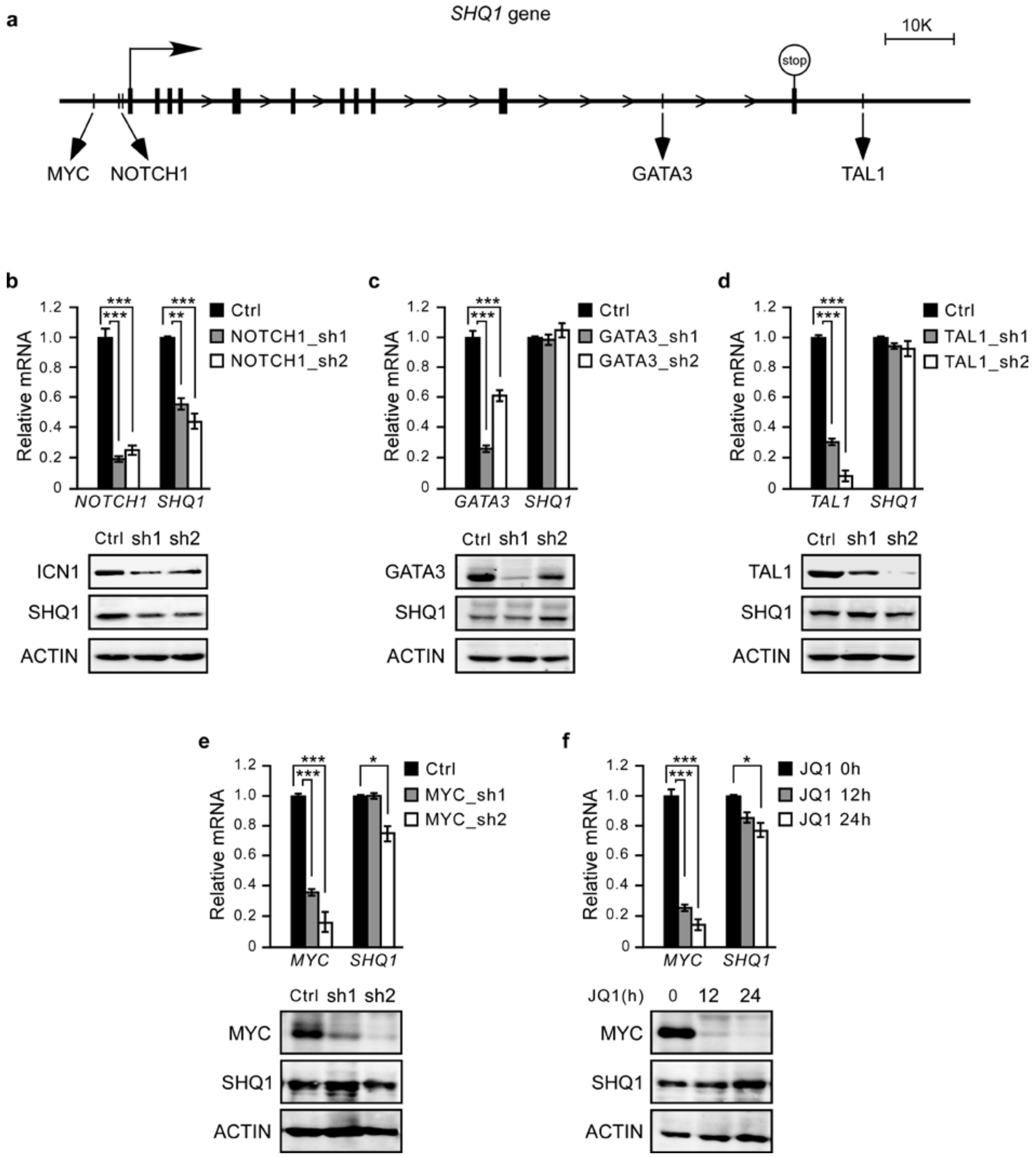

Supplementary Figure 1. SHQ1 expression is primarily regulated by NOTCH1 in T-ALL. a Schematic presentation of the *SHQ1* locus with tentative binding sites by transcriptional factors implicated in T-ALL. **b-f** JURKAT cells were transduced with indicated shRNAs for 48 h in (**b-e**) or treated with 250 nM JQ1 (**f**). *SHQ1* mRNA and protein levels were subsequently determined by qPCR and immunoblot. Data shown represent the means (±SEM) of 3 biological replicates. *p<0.05, **p<0.01, ***p<0.001, unpaired t test (**b-f**).

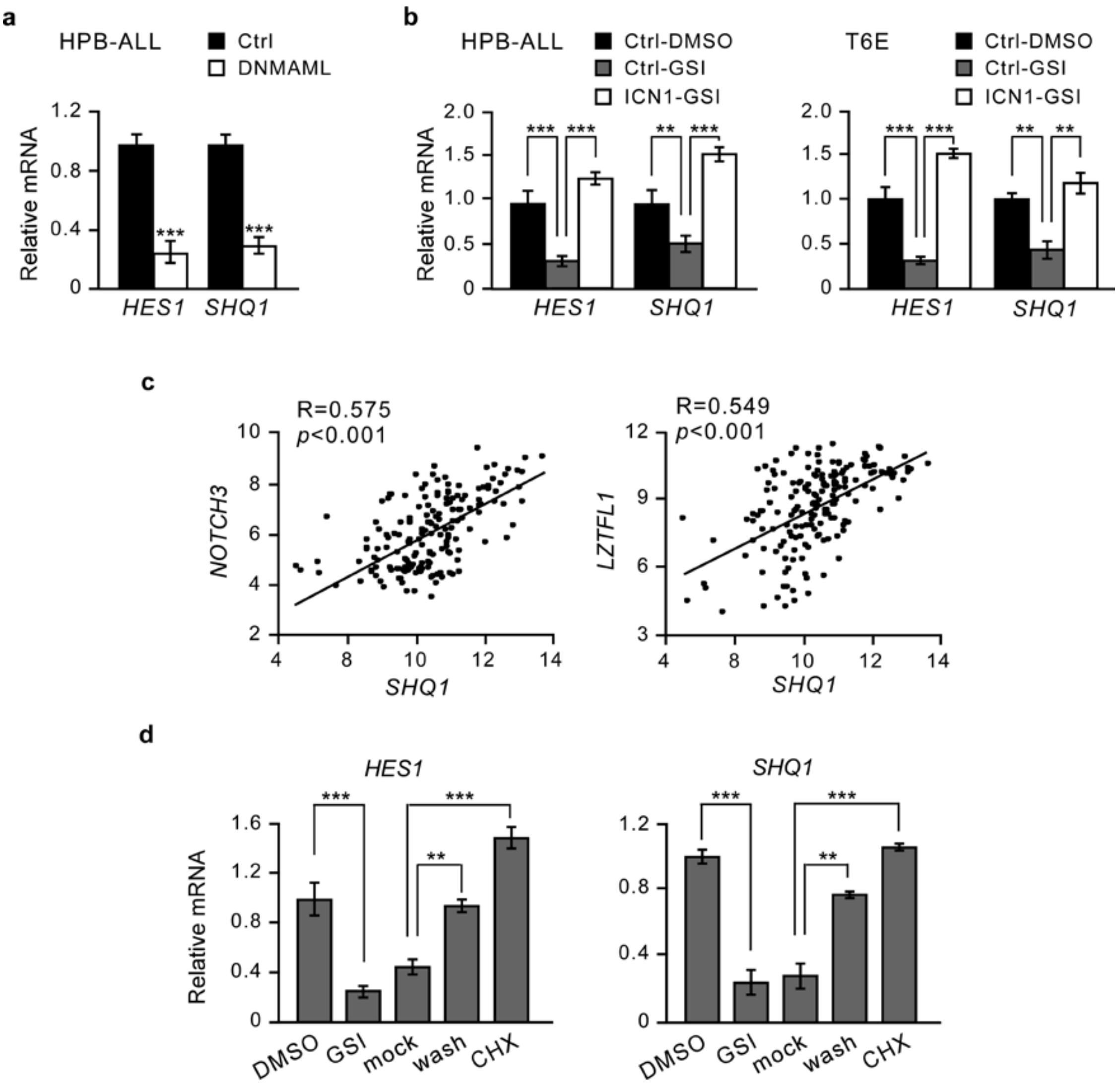

Supplementary Figure 2. NOTCH1 activates *SHQ1* transcription in T-ALL. a NOTCH activity was blocked in HPB-ALL cells by lentiviral expression of pCDH-DNMAML or pCDH (Ctrl) for 48 h. *SHQ1* and *HES1* mRNA were quantified by qPCR. **b** NOTCH1 activity was reconstituted by infection of pCDH-ICN1 in HPB-ALL or MigR1-ICN1 in T6E. Transductions of empty pCDH or MigR1 vectors are shown as control (Ctrl). Following DMSO or GSI (Compound E, 1 μM) treatment for 24 h, *SHQ1* and *HES1* mRNA were quantified by qPCR. **c** Correlation of *SHQ1* expression with previously reported NOTCH1-regulated signature genes (NOTCH3 or LZTFL1, ref. Roti G et al, Cancer Cell 2013) in 174 primary T-ALL samples (GSE13159) with mRNA levels presented as log2 median-entered intensity. The Pearson's correlation coefficient (R) and *p*-value (paired *t* test) are shown. **d** KOPTK1 cells were treated with GSI (Compound E, 1 μM) for 48 h, then washed and refed medium containing Compound E (mock), medium lacking Compound E (wash) or wash medium with cycloheximide (CHX, 100 μg mL^{-1}). *SHQ1* and *HES1* mRNA were quantified by qPCR after 6 h additional culture. Data shown represent the means (±SEM) of triplicates. **p<0.01, ***p<0.001, unpaired *t* test (**a**, **b**, **d**).

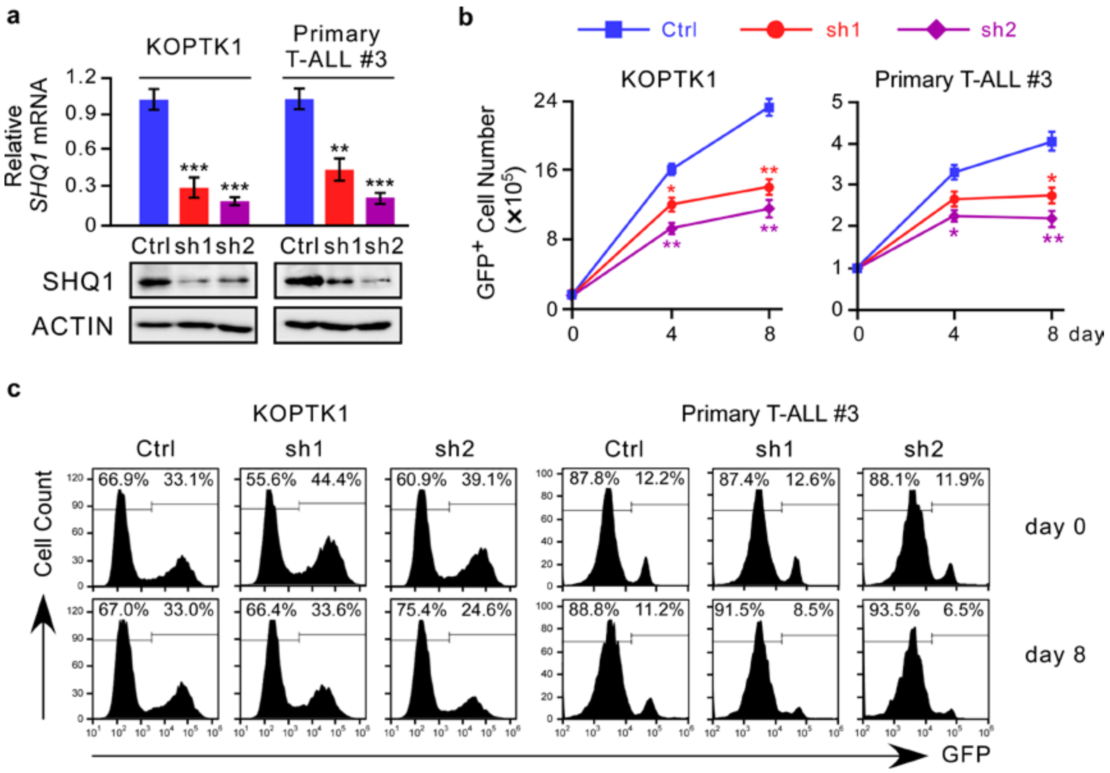

Supplementary Figure 3. *SHQ1* knockdown in T-ALL cells causes a severe growth disadvantage. a KOPTK1 cells and patient-derived primary T-ALL cells (#3) were infected with lentiviruses expressing control (Ctrl, blue) or *SHQ1* shRNA (sh1-red or sh2-purple). **b** Live GFP^+ KOPTK1 cells were counted and plotted. The growth assay was also conducted in patient-derived primary T-ALL cells (#3). Growth curves of T-ALL cells expressing control shRNA, *SHQ1* shRNA-1 or 2 are shown as blue, red or purple lines. **c** Percentages of GFP^+ cell populations were analyzed by flow cytometry at the indicated time points. Data shown represent the means (±SEM) of 3 biological replicates. *p<0.05, **p<0.01, unpaired t test (**a**, **b**).

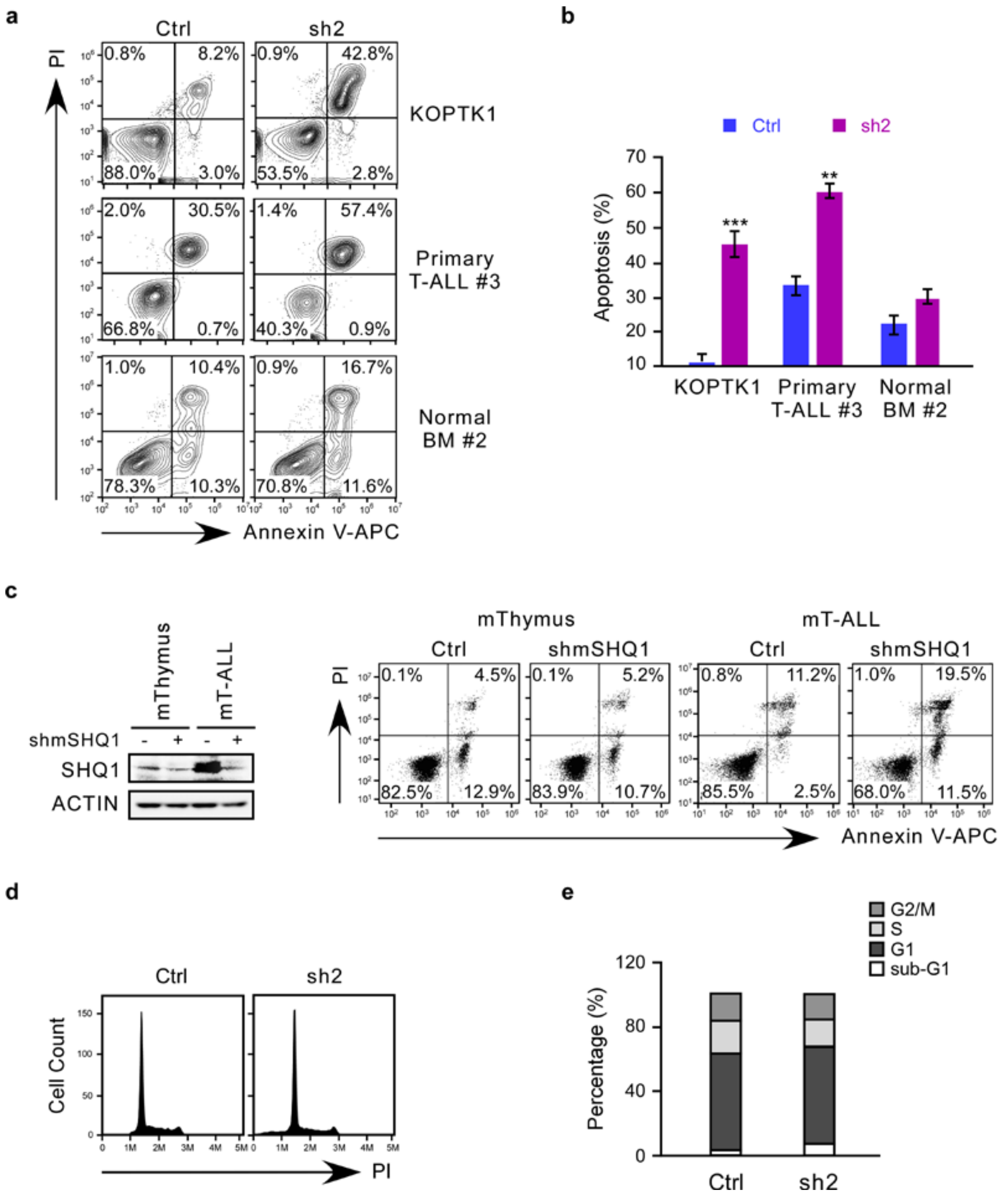

Supplementary Figure 4. ***SHQ1*** **ablation induces T-ALL cell apoptosis while imposes minimal effect on cell cycle. a-b** KOPTK1, primary T-ALL (#3) or normal BM (#2) cells were transduced with control shRNA (Ctrl) or *SHQ1* shRNA (sh2) viruses. Apoptotic cell death in shRNA-expressing cells was determined by Annexin V-PI staining 6 days post infection and quantified as shown in (**b**) (blue: control shRNA, purple: *SHQ1* shRNA-2). Data shown represent the means (±SEM) of 3 biological replicates. $^{**}p<0.01$, $^{***}p<0.001$, unpaired *t* test. **c** Murine T-ALL cell $Kras^{G12D}/NOTCH1^{L1601P}$ (mT-ALL) and normal murine thymus cells (mThymus) were transduced with control shRNA (Ctrl) or murine *SHQ1* shRNA (shmSHQ1) viruses. Apoptotic cell death in shRNA-expressing cells was determined by Annexin V-PI staining 6 days post infection. **d-e** Cell cycle was analyzed in HPB-ALL cells with or without *SHQ1* knockdown. Mean percentages of cells in sub-G1, G1, S and G2/M phase are presented in (**e**).

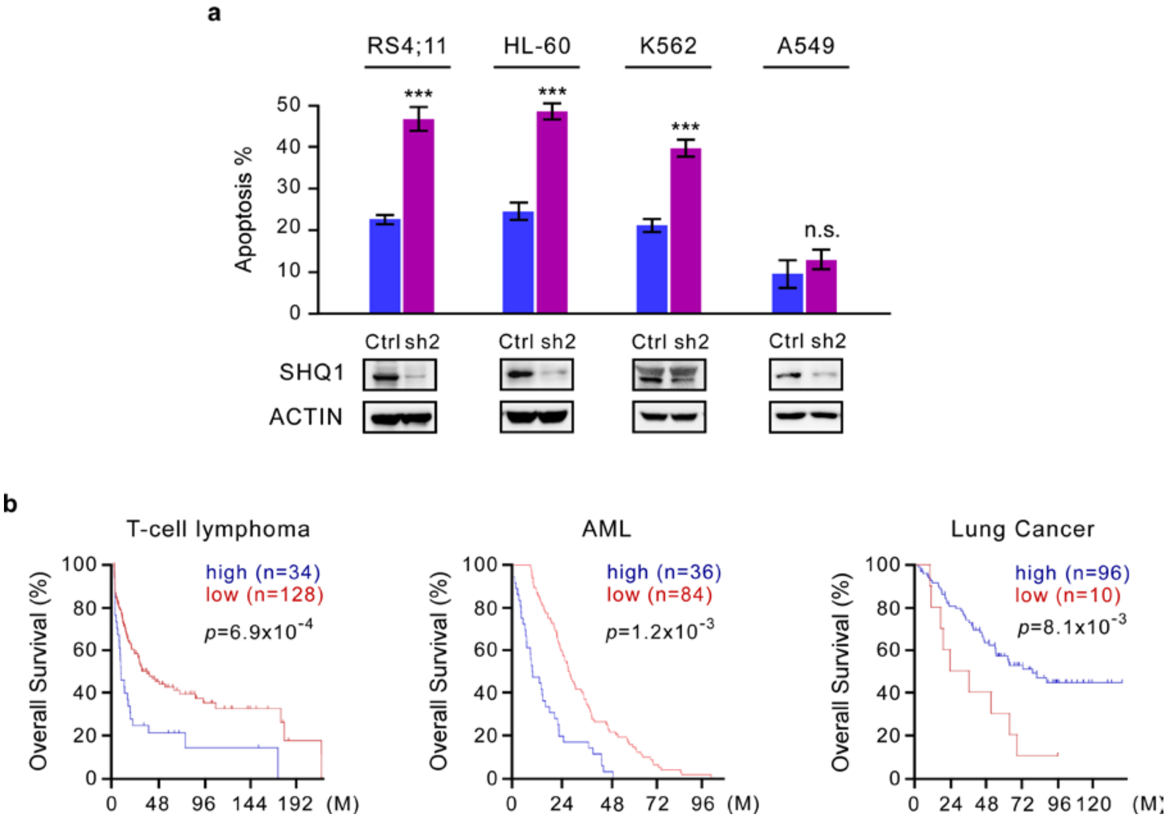

Supplementary Figure 5. SHQ1 plays differential roles in tumor cell survival and tumorigenesis. a RS4;11 (B-ALL), HL-60 (AML), K562 (CML) and A549 (lung cancer) cells were transduced with control shRNA (Ctrl, blue) or *SHQ1* shRNA-2 (sh2, purple) viruses. Apoptotic cell death in shRNA-expressing cells was determined by Annexin V-PI staining 6 days post infection and quantified as shown. Data shown represent the means (±SEM) of 3 biological replicates. ***p<0.001, n.s.: non-significant, unpaired t test. **b** Overall survival curves of patients with *SHQ1* high (blue) or low (red) expression in T-cell lymphoma, AML and lung cancer. The prognosis information was obtained from R2: Genomics Analysis and Visualization Platform [https://hgserver1.amc.nl/ cgi-bin/r2/main.cgi].

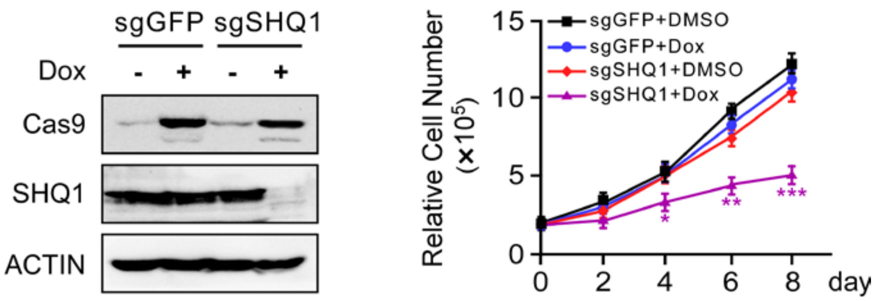

Supplementary Figure 6. *SHQ1* knockout in JURKAT cells results in cell growth arrest. JURKAT cells were infected with lentiviruses expressing control (GFP) or *SHQ1* sgRNA together with doxycycline inducible Cas9. After doxycycline treatment (1 μg mL^{-1}) for 3 days, Cas9 and SHQ1 expression were assessed by immunoblot (left). Cells were subsequently counted at the indicated time points and growth curves are depicted as shown on the right. (sgGFP+DMSO, black; sgGFP+Dox, blue; sgSHQ1+DMSO, red and sgGFP+Dox, purple). Data shown represent the means (±SEM) of 3 biological replicates. *p<0.05, **p<0.01, ***p<0.001, unpaired t test.

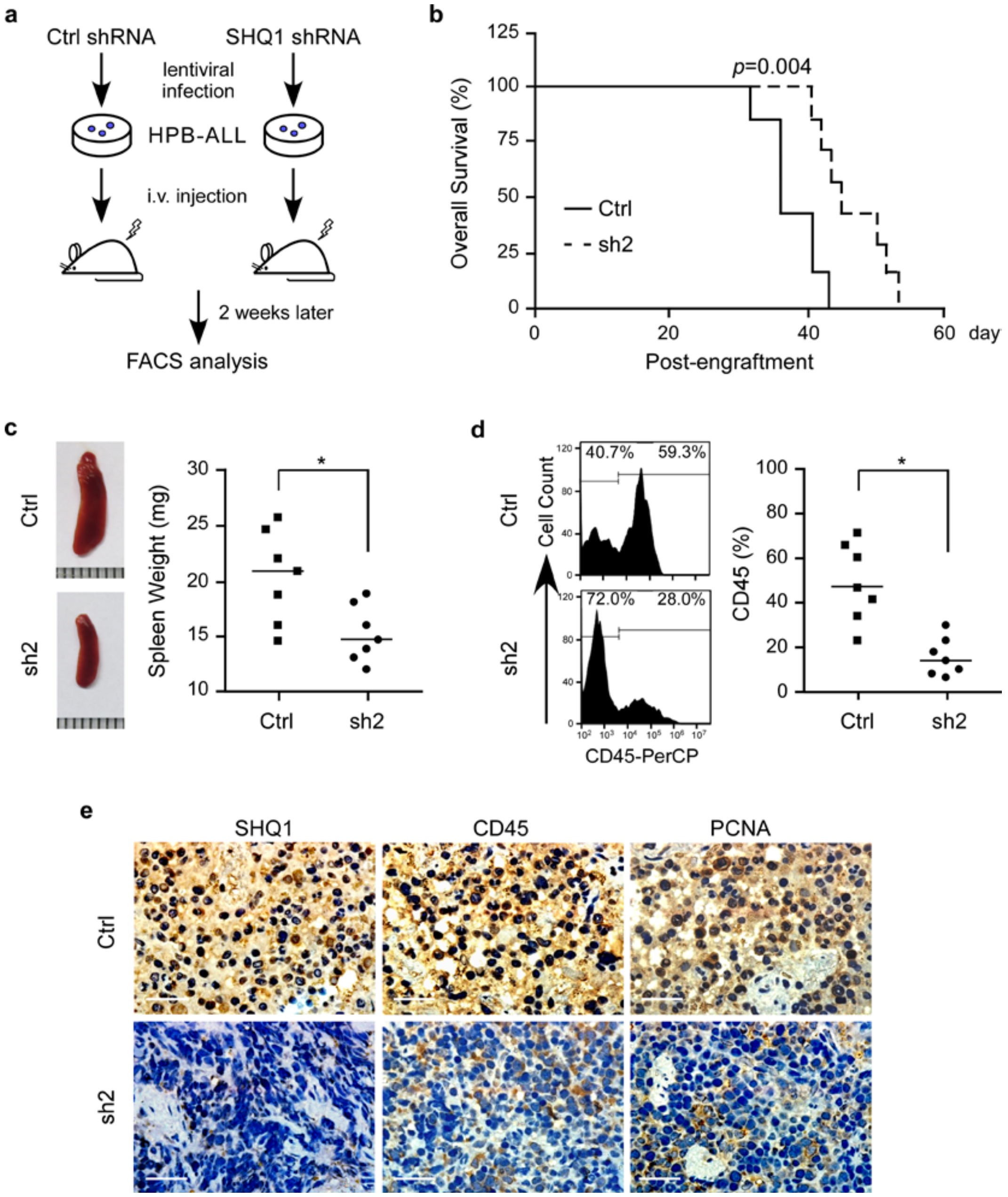

Supplementary Figure 7. *SHQ1* knockdown decreases T-ALL burden in human T-ALL xenograft. a Schematic representation of HPB-ALL xenografts. HPB-ALL cells expressing control or *SHQ1* shRNA were intravenously injected into irradiated NPG mice (1 Gray) four days post lentiviral infection. **b** Kaplan–Meier survival curves of mice with HPB-ALL cells having control shRNA (Ctrl, *n*=7) or *SHQ1* shRNA (sh2, *n*=7). **c** Representative spleen images (left) and spleen weights around 40 days post injection are plotted on the right. **d** Human CD45$^+$ cells from the spleen in (**c**), representing engrafted HPB-ALL cells, were analyzed by flow cytometry. Data from 7 mice in each group are shown on the right. *p<0.05, unpaired *t* test (**c, d**). **e** Representative immunohistological images of human SHQ1, CD45 and PCNA in spleen sections from the control and *SHQ1* shRNA mice. Scale bar, 50 μm.

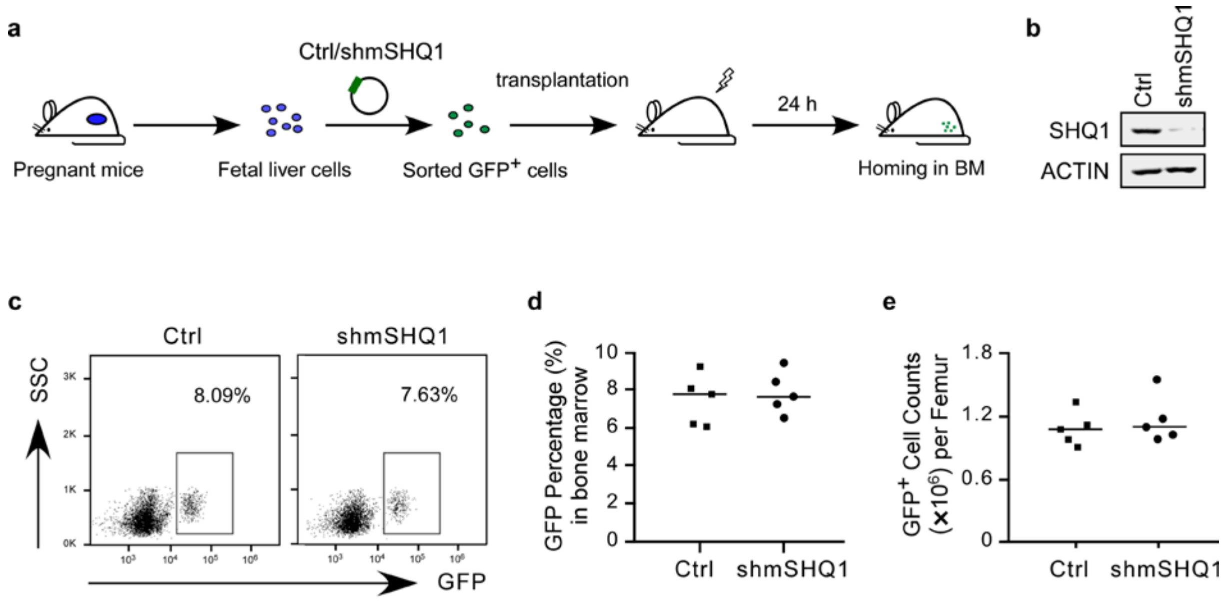

Supplementary Figure 8. *SHQ1* depletion does not impair hematopoietic stem/progenitor cell (HSPC) homing *in vivo*. a Schematic presentation of fetal liver cell transplantation and homing detection. GFP^+ fetal liver cells infected with retroviruses of *SHQ1* or control shRNA were sorted for transplantation. A total of 2 million GFP^+ cells were intravenously injected into lethally irradiated recipient mice (Ctrl: *n*=5, shmSHQ1: *n*=5), which were sacrificed 24 h post-transplantation. GFP^+ cells from bone marrow, representing HSPC homing to bone marrow, were analyzed by flow cytometry. **b** Murine *SHQ1* knockdown was confirmed by immunoblot, prior to transplantation, in sorted GFP^+ HSPCs expressing murine *SHQ1* shRNA. **c** Representative FACS images are shown depicting GFP^+ cell percentages in bone marrow of recipient mice. **d-e** GFP^+ percentages in recipient bone marrow (**d**) and absolute counts of GFP^+ cells per femur (**e**) in each group of mice were plotted.

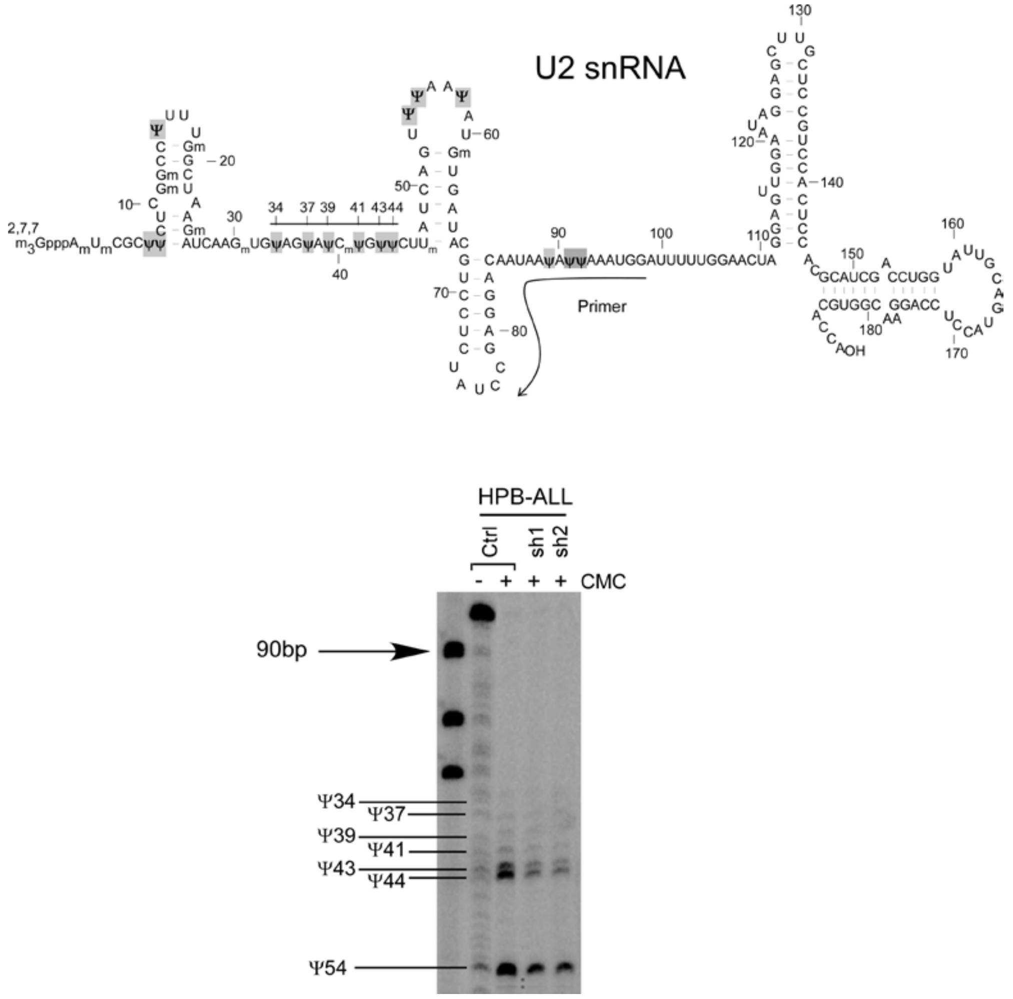

Supplementary Figure 9. *SHQ1* knockdown impairs pseudouridylation on U2 snRNA. The primary sequence and secondary structure of U2 snRNA are shown at the top. Total RNA isolated from control (Ctrl) and *SHQ1* shRNA (sh1 or sh2) infected HPB-ALL cells were used for U2 snRNA pseudouridylation assay — N-cyclohexyl-N-(2-morpholinoethyl)-carbodiimid-methop-toluolsulfonate (CMC) modification followed by primer extension. The CMC-modified Ψ residues in an RNA substrate would stop primer extension one nucleotide before the CMC-Ψ site (ref. Huang C et al, Methods 2016). Primer extension/pauses correspond to Ψ sites (indicated on the left in the bottom panel). The primer used for mapping Ψs in U2 snRNA was annealed to nucleotides 77-98 of U2, which was shown as curved line with arrow. Pseudouridylation of sites 34-44 is H/ACA RNPs dependent and this region is responsible for recognizing branch site of target pre-mRNA.

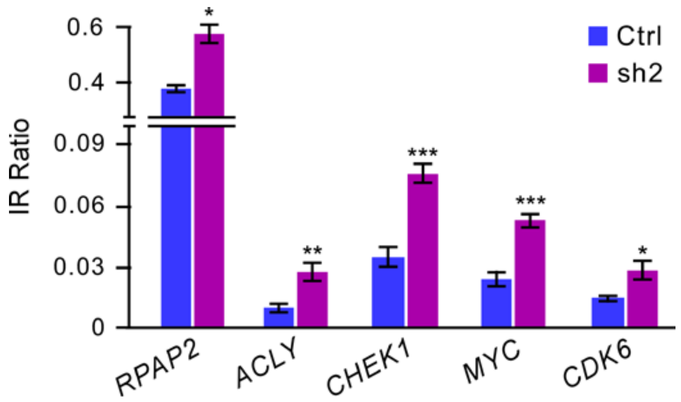

Supplementary Figure 10. Impaired candidate gene splicing in primary T-ALL with *SHQ1* loss. Candidate genes revealed in Fig. 6d were analyzed of splicing efficiency in primary T-ALL sample (#1) upon *SHQ1* depletion. Primary T-ALL cells were transduced with control (Ctrl, blue) or *SHQ1* shRNA-2 (sh2, purple) viruses. Intron retention (IR) ratios of *RPAP2*, *ACLY*, *CHEK1*, *MYC* and *CDK6* were assessed by qPCR using specific primer listed in Supplementary Table 1. Data shown represent the means (±SEM) of 3 biological replicates. *p<0.05, **p<0.01, ***p<0.001, unpaired t test.

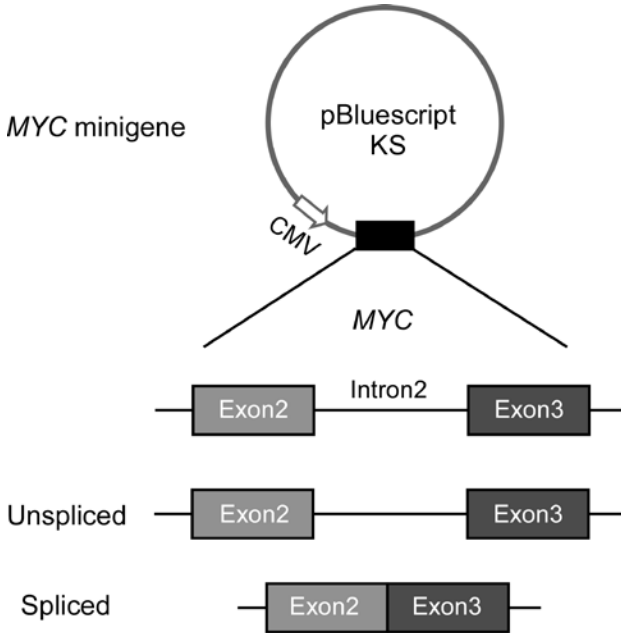

Supplementary Figure 11. Schematic of *MYC* minigene construct. The genomic fragments carrying *MYC* intron 2 and flanking exons on each side (exon 2 and 3) were amplified and cloned into the Nde I site in a modified pBluescript KS vector. Accurate orientation of the inserted DNA fragments was validated by DNA sequencing. This plasmid was used in minigene splicing assay in Fig. 7a and Supplementary Fig. 14.

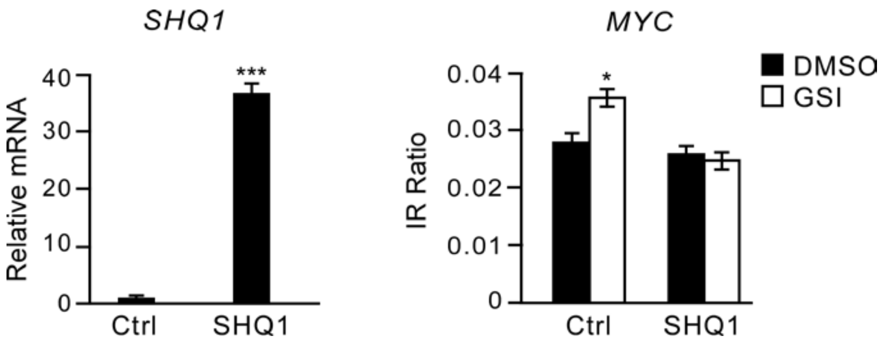

Supplementary Figure 12. Enforced *SHQ1* expression rescues NOTCH1 inhibition-mediated *MYC* splicing defect. HPB-ALL cells were transduced with pCDH-SHQ1 or pCDH vector (Ctrl) lentivirus. *SHQ1* overexpression was confirmed by qPCR (left). In the presence or absence of GSI (Compound E, 1μM), intron retention (IR) ratio of *MYC* was analyzed by qPCR using primer sets E2-E3 and E2-I2 shown in Fig. 7b. Data shown represent the means (±SEM) of 3 biological replicates. $*p<0.05$, $***p<0.001$, unpaired *t* test.

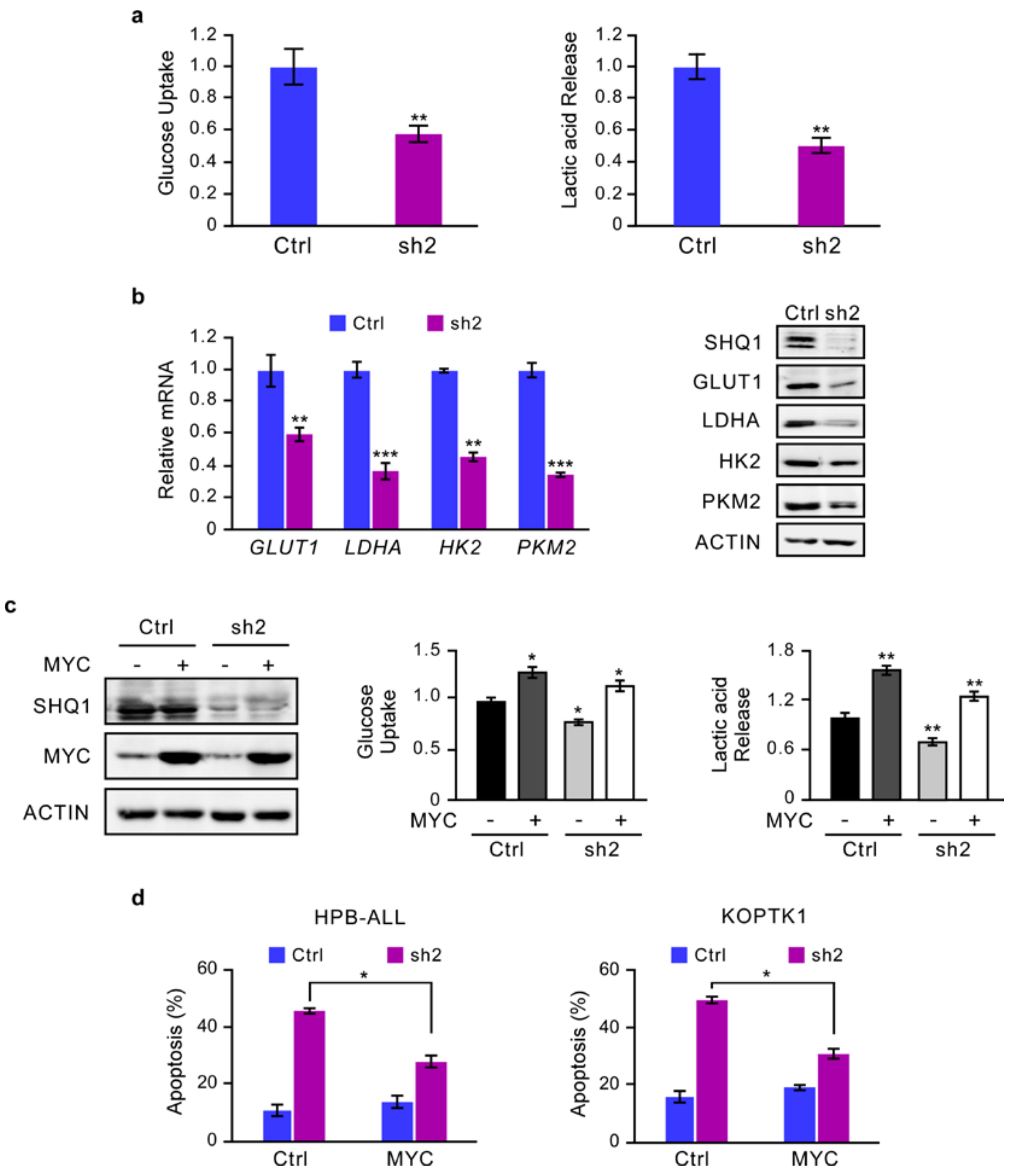

Supplementary Figure 13. Overexpression of MYC rescues attenuated glycolysis and cell death resulting from *SHQ1* loss. a KOPTK1 cells were infected with control (Ctrl, blue) or *SHQ1* shRNA-2 (sh2, purple) viruses. Glucose uptake and lactate secretion normalized to the same number of transduced live cells were examined 4 days post transduction. **b** Analysis of MYC target genes implicated in glycolysis by qPCR and immunoblot in control(Ctrl, blue) or *SHQ1*-depleted (sh2, purple) KOPTK1 cells. **c** As a supplementary data for Fig. 7f, HPB-ALL cells were co-infected with pCDH-MYC and *SHQ1* shRNA viruses as denoted in the left panel. Glucose uptake and lactate secretion normalized to the same number of transduced live cells were examined six days post infection. **d** As a supplementary data for Fig. 7g, assessment of apoptosis in HPB-ALL and KOPTK1 cells overexpressing MYC or not, depleted for *SHQ1* (sh2, purple) or not (Ctrl, blue) as indicated. Apoptotic cell death was detected using Annexin-V/ PI staining six days post infection. Data shown represent the means (±SEM) of 3 biological replicates. **p*<0.05, ***p*<0.01, ****p*<0.001, unpaired *t* test.

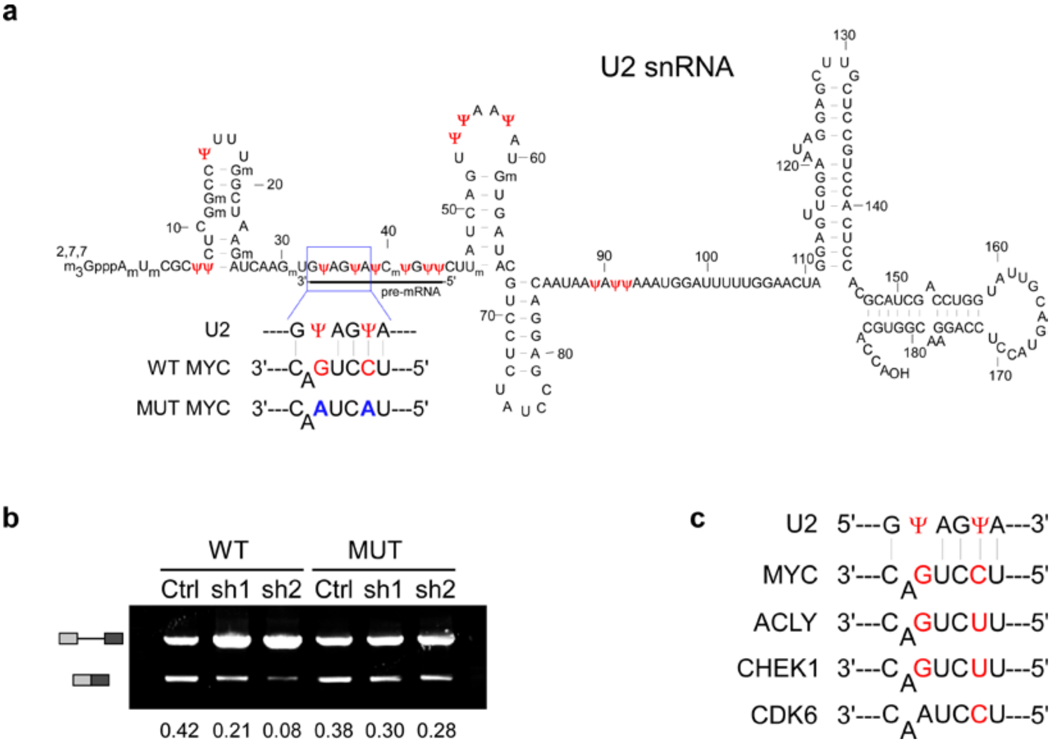

Supplementary Figure 14. Specific requirement of SHQ1 for *MYC* RNA splicing depends on a unique intronic sequence. a Schematic presentation of *MYC* pre-mRNA branch site (WT MYC) base paired with pseudouridines (Ψ) in U2 snRNA. The bulged-out nucleotide adenosine (A) that makes nucleophilic attack at the 5′ splice site in the first step of splicing. Nonconventional Ψ-G and Ψ-C are marked in red; mutant MYC branch site (MUT MYC) is shown at the bottom and mutated A is labeled in blue. The wild type and mutant MYC minigene constructs were used to perform the same splicing assay as the upper panel in Fig. 7a. **b** *MYC* minigene splicing was analyzed in 293T cells expressing control (Ctrl) or *SHQ1* shRNA (sh1 or sh2). Semi-quantitative RT-PCR was performed to analyze unspliced and spliced minigene RNA. Splicing efficiency was calculated and shown as the ratio of spliced vs total RNA. Unique Ψ-G and Ψ-C base pairs confer enhanced dependency on the activity of pseudouridylated U2 snRNA. **c** Intronic sequences similar to that found in *MYC* intron (**a**) were revealed in multiple other candidate genes from Fig. 6e-6f.

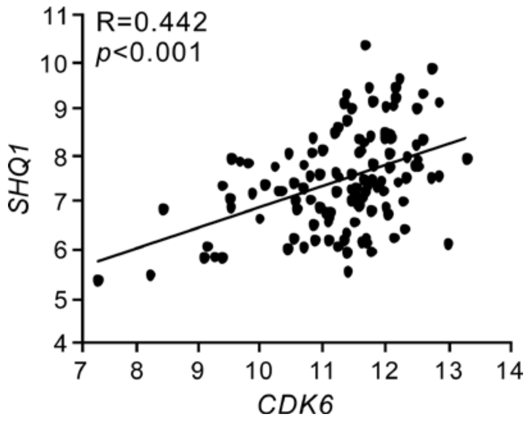

Supplementary Figure 15. Correlation of *SHQ1* with *CDK6* mRNA in 117 primary T-ALL samples (GSE26713). Pearson's correlation coefficient (R) and *p*-value are shown.

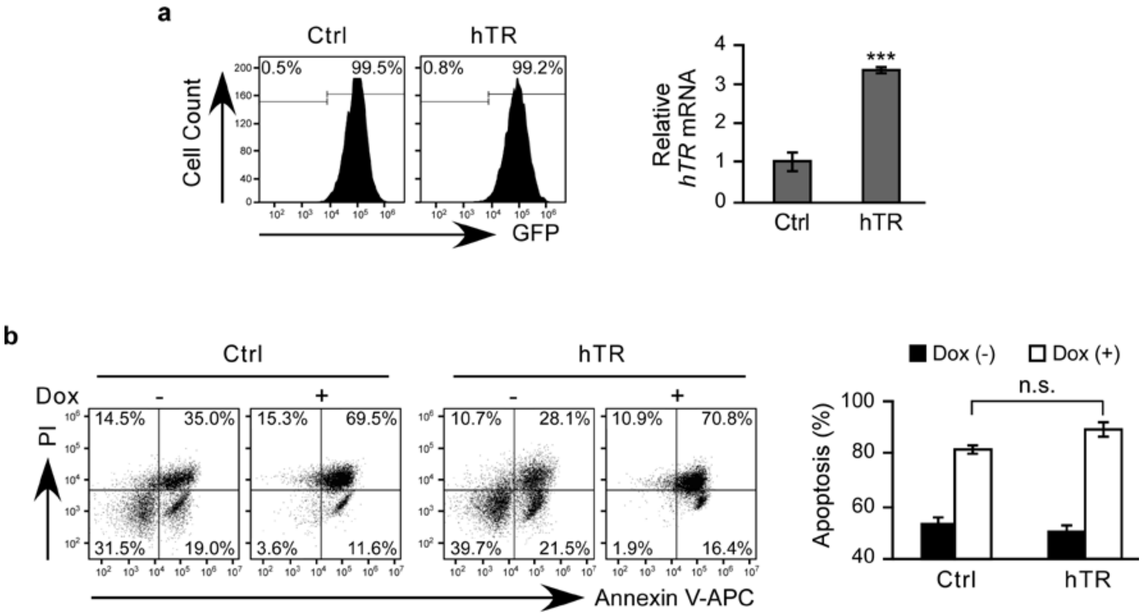

Supplementary Figure 16. Enforced hTR expression fails to rescue *SHQ1* depletion-induced cell apoptosis. a Inducible *SHQ1* knockout JURKAT cells were transduced with pCDH vector alone (Ctrl) or pCDH-hTR (hTR). hTR expression were analyzed in selected GFP^+ cells by qPCR. **b** JURKAT cells were subjected to doxycycline (1 μg mL^{-1}) treatment to induce *SHQ1* knockout. Apoptotic cell death was assessed by Annexin V-PI staining and flow cytometry analysis. Percentages of apoptotic cells were graphed and shown on the right. Data shown represent the means (±SEM) of 3 biological replicates. ***p<0.001, n.s.: non-significant, unpaired t test.

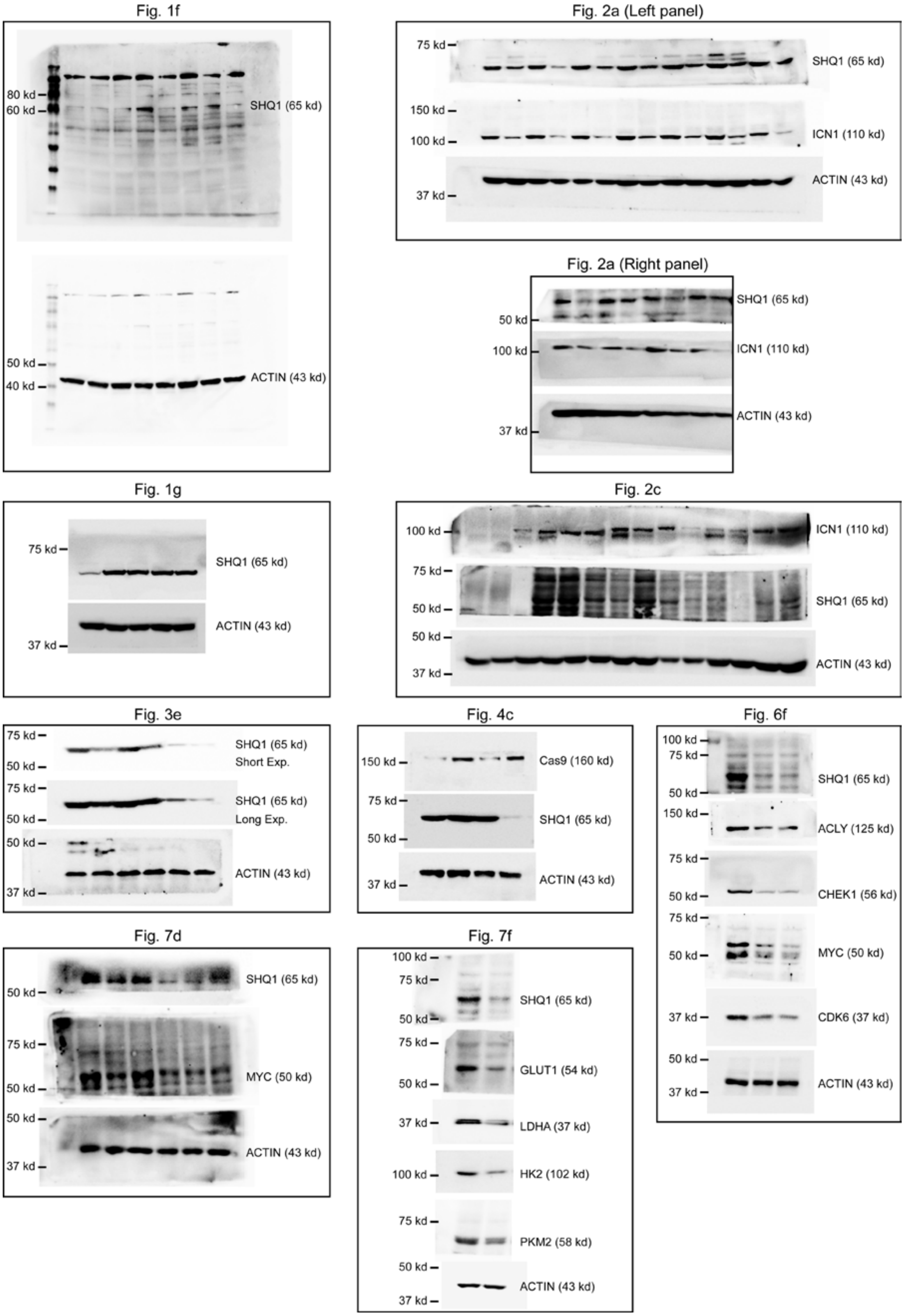

Supplementary Figure 17. Uncropped images of immunoblots shown in Figures 1-7 with indicated molecular weight markers.

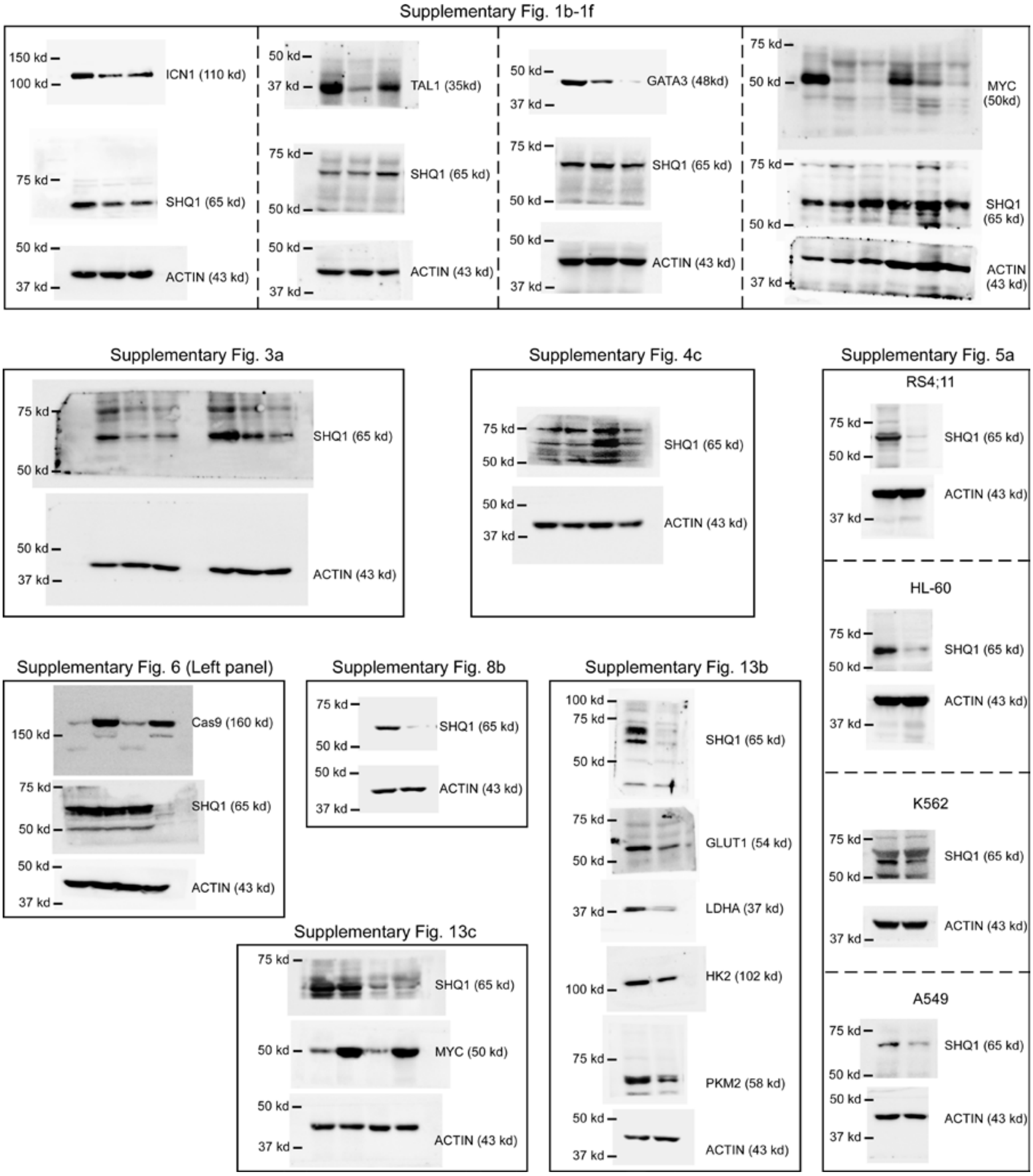

Supplementary Figure 18. Uncropped images of immunoblots shown in the Supplementary Figures with indicated molecular weight markers.

Supplementary Table 1. List of primers used in this study. Human genes are detected unless otherwise indicated.

**Primers for real-time PCR**	
ACTIN forward	5′-CACCATTGGCAATGAGCGGTTC-3′
ACTIN reverse	5′-AGGTCTTTGCGGATGTCCACGT-3′
SHQ1 forward	5′-GCTTGTCCAGGAGGAAGAAAC -3′
SHQ1 reverse	5′-CTTCGTTTCCAGATGACACGC-3′
HES1 forward	5′-TCAACACGACACCGGATAAA-3′
HES1 reverse	5′-TCAGCTGGCTCAGACTTTC-3′
scaU93 forward	5′-ATCTGTAGTCTTGGAGCCGC-3′
scaU93 reverse	5′-GTGGCAACAGTGACCAGAAAC-3′
RPAP2 E8 forward	5′-CAGAGGACGGTATGTTTTGGGTG-3′
RPAP2 I8 reverse	5′-GCCAACAGTGGAGATGGCAC-3′
RPAP2 E9 reverse	5′-TTCTGGGAACTTGAGTCTATCAGTGG-3′
ACLY E1 forward	5′-CGCACGGACTTCGGCAGAG-3′
ACLY I2 forward	5′-AGGCAGGAAGCTCCTAGGGC-3′
ACLY E2 reverse	5′-GTGTCAGGAGTGACCCGAGC-3′
CHEK1 E3 forward	5′-GGAGTACTGTAGTGGAGGAGAGC-3′
CHEK1 I3 reverse	5′-GACAACGACCAAATAGCATGCCC-3′
CHEK1 E4 reverse	5′-CACCCCTGCCATGAGTTGATG-3′
CDK6 E2 forward	5′-CATGCCGCTCTCCACCATCC-3′
CDK6 I2 reverse	5′-CTGGGCCTGAGGATTCCCG-3′
CDK6 E3 reverse	5′-GTTCGTGACACTGTGCACACATC-3′
MYC E1 forward	5′-GGGAGGCTATTCTGCCCATT-3′
MYC I1 reverse	5′-TGGACTTCGGTGCTTACCTG-3′
MYC E2 reverse	5′-AGCTAACGTTGAGGGGCATC-3′
MYC E2 forward	5′-CCTACCCTCTCAACGACAGC-3′
MYC I2 reverse	5′-GGGCTTCGCTTACCAGAGTC-3′
MYC E3 reverse	5′-TGTTCCTCCTCAGAGTCGCT-3′
NOTCH1 forward	5′-GCAGAGGCGTGGCAGACTA-3′
NOTCH1 reverse	5′-ACGATTTCCCTGACCAGCCG-3′
AKT1 forward	5′-CAGCCTGGGTCAAAGAAGTCA-3′
AKT1 reverse	5′-GATGTACTCCCCTCGTTTGTG-3′
TAL1 forward	5′-TCCGTTGGTGTTCTCAGCAG-3′
TAL1 reverse	5′-CGGTCATCCTGGGGCATATT-3′
LMO2 forward	5′-GGACCCTTCAGAGGAACCAG-3′
LMO2 reverse	5′-CTTCAGGAAGTAGCGGTCCC-3′
GATA3 forward	5′-GCACGGGACACTACCTGTGC-3′
GATA3 reverse	5′-CTCTCCTGGCTGCAGACAGC-3′
hTR forward	5′-CGCTGTTTTTCTCGCTGACTT-3′
hTR reverse	5′-TGCTCTAGAATGAACGGTGGAA-3′
murine *ACTIN* forward	5′-GGCTGTATTCCCCTCCATCG-3′
murine *ACTIN* reverse	5′-CCAGTTGGTAACAATGCCATGT-3′
murine *SHQ1* forward	5′-GCTAGACAAGACGGCTCACC-3′
murine *SHQ1* reverse	5′-ATTCAACGCTGTGCTCTCCT-3′
murine *HES1* forward	5′-GCACAGAAAGTCATCAAAGCC-3′
murine *HES1* reverse	5′-ATGCCGGGAGCTATCTTTCT-3′

GLUT1	TaqMan probe, Applied Biosystems, Hs00892681_m1
LDHA	TaqMan probe, Applied Biosystems, Hs01378790_g1
HK2	TaqMan probe, Applied Biosystems, Hs00606086_m1
PKM2	TaqMan probe, Applied Biosystems, Hs00761782_s1
**Primers for ChIP assay**	
ACTIN promoter forward	5'-GACTTCTAAGTGGCCGCAAG-3'
ACTIN promoter reverse	5'-TTGCCGACTTCAGAGCAAC-3'
SHQ1 promoter forward	5'-TAGGTTCTCGCATTATCCGC-3'
SHQ1 promoter reverse	5'-GATGACTAGCAACCGAGCC-3'
HES1 promoter forward	5'-CGTGTCTCCTCCTCCCATT-3'
HES1 promoter reverse	5'-CGGATCCTGTGTGATCCCTA-3'
**Primers for luciferase reporter constructs**	
SHQ1 promoter WT forward	5′-GGTACCGGAATTGTTCACAAGGCAGGTG-3′
SHQ1 promoter WT reverse	5′-CGCTCGAGGCTGTAAGACCTTGGGAAAGTTAC-3′
SHQ1 promoter mutant forward	5′-GGTACCGGAATTGAGCACAAGGCAGGTG-3′
SHQ1 promoter mutant reverse	5′-CGCTCGAGGCTGTAAGACCTTGGTGCAGTTAC-3′
**Primers for cloning**	
pCDH-MYC forward	5′-CGGAATTCGCCACCATGCCCCTCAACGTTAGCTTC-3′
pCDH-MYC reverse	5′-CGGGATCCTTACGCACAAGAGTTCCGTAGC-3′
pCDH-SHQ1 forward	5′-ATGCTCTAGAGCCACCATGCTGACCCCGGCGTTC-3′
pCDH-SHQ1 reverse	5′-ATGCGAATTCTCAATTATTTGGTGTCTGACAGCCG-3′
pCDH-hTR forward	5′-CGGAATTCGGGTTGCGGAGGGTGGG-3′
pCDH-hTR reverse	5′-CGGGATCCGCATGTGTGAGCCGAGTCCTG-3′
MYC minigene WT forward	5′-GGGAATTCCATATGGTCACACCCTTCTCCCTTCG-3′
MYC minigene WT reverse	5′-GGGAATTCCATATGGCTGCGTAGTTGTGCTGATG-3′
MYC minigene mutant forward	5′-GGATGGTCTCTACTAACCTCACGATCCGCC-3′
MYC minigene mutant reverse	5′-GGCGGATCGTGAGGTTAGTAGAGACCATCC-3′
**shRNA sequences**	
SHQ1_sh1	5′-CCTGAGGAAGTAGTTGACGAT-3′
SHQ1_sh2	5′-GCCAAGTTTGATCCTGATCAT-3′
NOTCH1_sh1	5′-GCATGTGTAACATCAACATCG-3′
NOTCH1_sh2	5′-CGCTGCCTGGACAAGATCAAT-3′
GATA3_sh1	5′-GCCTACATGCTTTGTGAACAA-3′
GATA3_sh2	5′-CATCCAGACCAGAAACCGAAA-3′
TAL1_sh1	5′-GCTCAGCAAGAATGAGATCCT-3′
TAL1_sh2	5′-AAGAAGCTCAGCAAGAATGAG-3′
MYC_sh1	5′-CAGTTGAAACACAAACTTGAA-3′
MYC_sh2	5′-CCTGAGACAGATCAGCAACAA-3′
murine shmSHQ1	5′-CTCAAGTGGTGCGCTCATTAC-3′
**sgRNA target sequences**	
sgSHQ1	5′-GGAAGTCCGGATCCTGGCTG-3′
sgGFP	5′-GGGCGAGGAGCTGTTCACCG-3′
