# Supplementary material for: SHQ1 regulation of RNA splicing is required for T-lymphoblastic leukemia cell survival
Source: Nat Commun. 2018 Oct 15;9:4281. doi: 10.1038/s41467-018-06523-4 (PMC6189109; doi:10.1038/s41467-018-06523-4)
Supplement: Supplementary file 2 — Description of Additional Supplementary Files [file 41467_2018_6523_MOESM2_ESM.doc]

**Description of Additional Supplementary File**

**Supplementary Data 1:** Common genes with elevated expression in 117 pediatric T-ALL samples (GSE26713).
